# Wafer-Level Self-Assembly and Interface Passivation Patterning Technology for Nanomaterial-Compatible 3D MEMS Sensing Chips

**DOI:** 10.1007/s40820-026-02080-4

**Published:** 2026-01-26

**Authors:** Zheng Zhang, Yanlin Zhang, Yuanyuan Luo, Guoliang Lv, Jianglin Yin, Pengwei Tan, Guotao Duan

**Affiliations:** 1 School of Integrated Circuits, Huazhong University of Science and Technology, Wuhan, 430074 People’s Republic of China; 2 Key Laboratory of Materials Physics, Institute of Solid State Physics, HFIPS, Chinese Academy of Sciences, Hefei, 230031 People’s Republic of China; 3 Wuhan National Laboratory for Optoelectronics, Huazhong University of Science and Technology, Wuhan, 430074 People’s Republic of China

**Keywords:** Compatible manufacturing, Wafer-level self-assembly, Interface passivation patterning technology, MEMS, Sensing chips

## Abstract

Wafer-scale fabrication of high-performance micro-electro-mechanical systems (MEMS) bio/chemical sensing chips remains constrained by the absence of reliable methods for integrating high-performance nanomaterials into suspended MEMS architectures. Here, a wafer-level manufacturing strategy is presented that redefines the MEMS process flow as “film first, cantilever later.” Through kinetically controlled self-assembly, wet-chemically synthesized Pd/SnO_2_ nanospheres are transferred as dense, uniform monolithic films onto 8-inch wafers. An HfO_2_ interface passivation patterning technology resolves long-standing incompatibility between functional sensing films and silicon substrates, enabling precise patterning and reliable integration on suspended MEMS cantilevers. The resulting Pd/SnO_2_ MEMS H_2_ chips are fabricated onto an 8-inch wafer, demonstrating high sensitivity and consistency. This approach overcomes long-standing wafer-level manufacturing challenges in the formation and patterning of high-performance nanomaterials film, establishing a fully integrated wafer-level process that fundamentally redefines the manufacturing route for tetramethylammonium hydroxide-resistant nanomaterial-based MEMS sensing chips.

**Supplementary Information:**

The online version contains supplementary material available at 10.1007/s40820-026-02080-4.

## Highlights

- Wafer-scale, kinetically controlled self-assembly combined with edge-controlled lift-off enables uniform and precisely patterned nanomaterial films on 8-inch wafers.
- HfO_2_ interface passivation eliminates wet etching failures and ensures reliable integration with suspended microelectro-mechanical systems (MEMS) structures.
- A “film-first, cantilever-later” strategy realizes 3D MEMS gas sensing chips with accurate nanomaterial incorporation, delivering high H_2_ sensitivity and uniformity.

**Supplementary Information:**

The online version contains supplementary material available at 10.1007/s40820-026-02080-4.

## Graphical abstract

**Figure:**
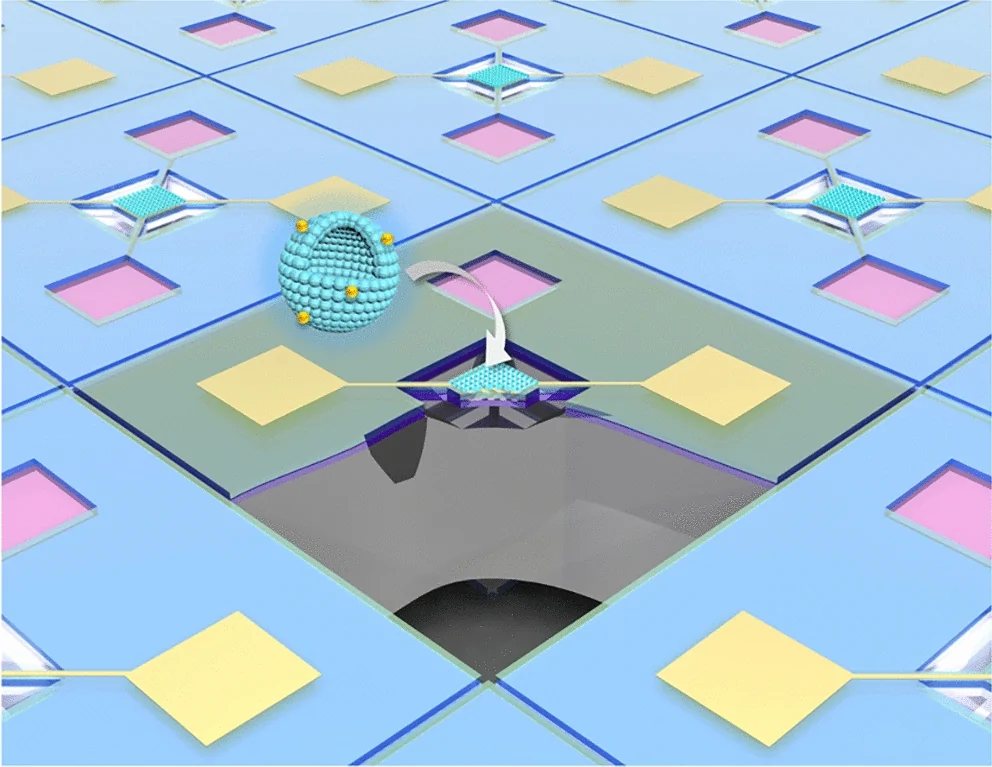

## Introduction

Micro-electro-mechanical systems (MEMS) serve as a cornerstone technology within the broader “More than Moore” paradigm, integrating diverse sensing and actuation functions to extend the capabilities of silicon-based platforms [1, 2]. Their inherent advantages—compactness, low cost, and energy efficiency—make MEMS sensors particularly promising for next-generation smart sensing applications [3–6]. Within this domain, bio/chemical sensors, such as micro-hotplates [7–10], micro-cantilevers [11, 12], and membrane-type surface stress sensors [13], have demonstrated strong application potential for gases, biomolecules, and chemical species detection.

High-performance bio/chemical sensors typically rely on sensitive nanomaterials with large surface-to-volume ratios, tunable crystal structures, and facile doping or surface functionalization [14–16]. Nevertheless, integrating such nanomaterials into suspended MEMS structures remains a long-standing challenge [17, 18], particularly in a manner compatible with standard wafer-level MEMS fabrication processes such as tetramethylammonium hydroxide (TMAH) cantilever release [19]. The key difficulties lie in converting initial powder-state nanomaterials into wafer-scale uniform films, and more importantly, achieving reliable film transfer and patterning on suspended structures. The suspended MEMS structures make lithography unusable because the photoresist coating collapses into the cavity and destroys the pattern. It also introduce gaps, causing the films to crack, delaminate, or spill into the cavity, degrading integrity and uniformity during transfer.

For micro-hotplate gas sensing chips, conventional post-transfer approaches apply nanomaterials onto pre-fabricated diced chips [20], resulting in low throughput, poor spatial accuracy, and thickness non-uniformity [21, 22] (Fig. S1), making them unsuitable for wafer-level manufacturing. Recent MEMS-compatible film deposition strategies have attempted to address these challenges. Self-lithographic deposition [23, 24] enables localized film formation, while mask-assisted patterned deposition [25, 26] can localize films on MEMS substrates. Electrospinning-based methods [27] offer controllable placement, and in situ synthesized films [28] can form continuous layers. Non-silicon nanotube array structures [29] also demonstrate excellent sensing performance. These methods each contribute important progress toward scalable integration of nanomaterials. However, they typically address only specific aspects of wafer-scale integration but do not yet provide a unified route that achieves MEMS-compatible wafer-level film formation, localized deposition, and tunable nanomaterial performance.

**Fig. 1:**
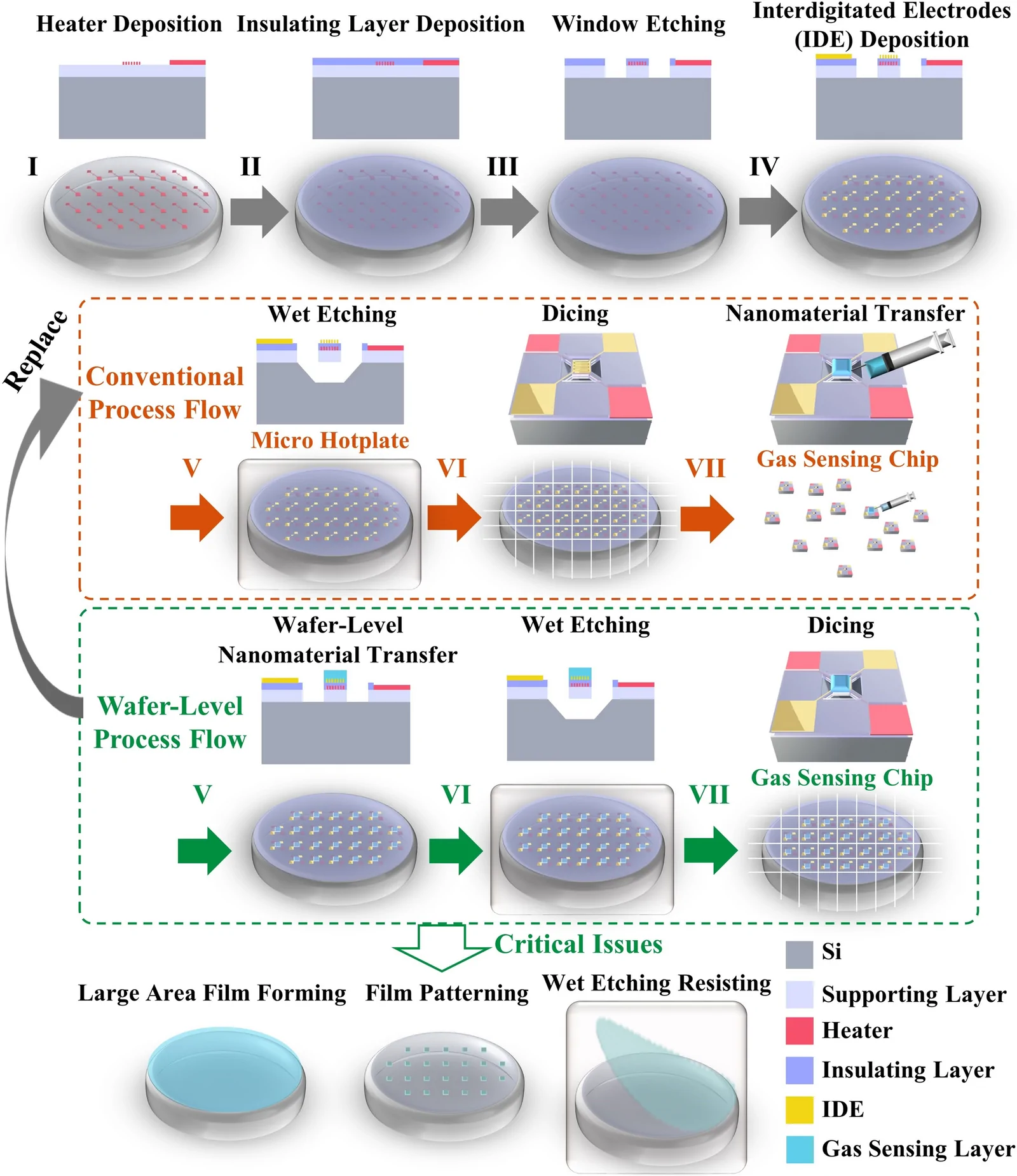
Comparison between conventional and wafer-level fabrication processes for MEMS gas sensing chips, along with associated challenges. Schematic flow of the conventional process flow: (I) Ti/Pt heater is patterned and deposited on a Si wafer with SiO_2_-Si_3_N_4_-SiO_2_ supporting films. (II) A SiO_2_ insulating layer is deposited. (III) Etching windows and heater pads are exposed. (IV) Ti/Pt interdigital testing electrodes are deposited. (V) Suspended cantilever is released to form a MEMS hotplate. (VI) Dicing to obtain separated micro-hotplate chips. (VII) Nanomaterials are manually transferred onto each device individually. In the wafer-level process, the final three steps are replaced (green arrows). (V) Wafer-level transfer of nanomaterials onto all devices simultaneously. (VI) Suspended cantilever is released to yield complete wafer-level MEMS gas sensing chips. (VII) Dicing. The bottom schematic illustrates three major challenges inherent to the wafer-level manufacturing proposal

This work presents a wafer-level “film-first, cantilever-later” process to replace conventional post-transfer strategy for incorporating high-performance TMAH-resistant nanomaterials into suspended MEMS structures (Fig. 1). A customized wafer-level self-assembly setup first converts nanomaterials into uniform monolayer films. By carrying out nanomaterial patterning on a planar wafer before the wet etching step, enabling the use of standard lithography and lift-off. Because the sensing film must tolerate alkaline wet etching during cantilever release, the failure mechanisms are investigated and an ALD-HfO_2_ passivation layer is introduced to protect the porous nanomaterial film throughout wet etching. The resulting process enables high-yield, consistent fabrication of nanomaterial-based MEMS sensing chips on an 8-inch wafer and is validated through the realization of hydrogen (H_2_) sensors. Overall, this work establishes a fully integrated wafer-level process that fundamentally redefines the manufacturing route for TMAH-resistant nanomaterial-based MEMS sensing chips.

## Experimental Section

### Nanomaterial Synthesis

SnO_2_ nanospheres were synthesized by a one-step hydrothermal method based on previous work [30]. 55 mL ethanol and deionized (DI) water (v/v = 10:1) mixture was first prepared, and then 0.8 g SnCl_4_ was dissolved, and 0.8 mL 35 wt% HCl was added into it with 30 min stirring. The mixture solution was loaded 12 h at 200 °C into hydrothermal autoclave reactors. Finally washing (by DI water and ethanol) and annealing (2 h at 400 °C in the air) to form SnO_2_ nanospheres. Pd/SnO_2_ was prepared based on the above nonannealing materials. 1.12 mg Pd(NH_3_)_4_Cl_2_·H_2_O and 300 mg materials were put into 200 mL DI water with 48-h stirring. After washing and drying, Pd-decorated materials were annealed in air for 5 h at 800 °C to form Pd/SnO_2_.

### SnO_2_ or Pd/SnO_2_ Suspension and Self-Assembly Film

70 mg SnO_2_ or Pd/SnO_2_ was added to 10 mL ethanol and 1 mL 1-dodecanethiol and then ultrasonically dispersed for 30 min and stirred for 48 h. After washing and drying, the materials could be used to prepare suspension. The ethanol suspension concentration is 30 mg L^−1^. Before self-assembly, the injection angle was adjusted perpendicular to the liquid level by a leveling device. Then put the wafer in, and add DI water to drown the whole wafer (the liquid level is 1 cm higher than the wafer surface). The automatic syringe was opened to inject suspension with 0.27 mL min^−1^ rate, and the film was gradually formed on the surface (285 s). After that, the controlling device of the liquid level was turned on to decrease the liquid level at the rate of 5 mL min^–1^ for film transfer. Self-assembly monolayer film was prepared under this process, and trilayer film refers to the process being repeated three times. The various patterned lithography was performed by a maskless direct writing system (MicroLab III).

### Density Functional Theory Calculation

All density functional theory (DFT) calculations were performed employing the Perdew–Burke–Ernzerhof form of generalized gradient approximation (GGA) and projector augmented wave (PAW) method, as implemented in the Vienna ab initio simulation package (VASP) code. Pseudopotentials with configurations of 2*s*^2^2*p*^4^, 5*d*^2^6*s*^2^_**,**_ and 3*s*^2^3*p*^2^ are used for O, Hf, and Si, respectively. DFT + *U* method was adopted for the strong Coulomb correlation of the Hf atom 5*d* electrons, where *U*_d_ are Hubbard correction of *d* orbitals and the values of *U*_d_ applied to Hf atom 5*d* electrons were set as 8 eV [31]. Amorphous HfO_2_ and SiO_2_ structures were obtained using Ab initio molecular dynamics (AIMD) simulations, incorporating annealing, cooling, and relaxation processes. Structural relaxations were performed with a convergence threshold of 1.0 × 10^–5^ eV atom^−1^ for electronic self-consistent iterations and 0.02 eV Å^−1^ for atomic forces. A plane-wave cutoff of 500 eV was used, and the Brillouin zone was sampled using the Monkhorst–Pack method with 3 × 3 × 1 mesh of k-point for stacked structures, respectively. Adsorption energies (ΔE_ads_) were defined as: ΔE_ads_ = E_adsorbate/slab_—(E_slab_ + E_adsorbate_), where E_adsorbate/slab_ is the total energy of the surface with the adsorbed H_2_O molecule or OH species and E_slab_ and E_adsorbate_ are the energies of stacked structure surface (HfO_2_ and SiO_2_ surface for HfO_2_/SiO_2_/Si and SiO_2_/Si structure, respectively) and the isolated adsorbate.

### MEMS Sensing Chip Fabrication

(i) 200–500-1100 nm SiO_2_-Si_3_N_4_-SiO_2_ supporting films were prepared on the N-type < 100 > Si substrate. (ii) The heater consists of 10/200 nm Ti/Pt was defined by lithography (MA8/BA8 GEN4) and deposited by RF magnetron sputtering (JCPY600). (iii) A 500 nm SiO_2_ insulating layer was deposited by plasma-enhanced chemical vapor deposition (Oxford PlasmaPro 800 Stratum PECVD) for insulating electrical signals, and (iv) then the 50c (~ 10.5 nm) HfO_2_ protective layer was prepared by ALD (precursors are Tetrakis(dimethylamido)hafnium(IV) and H_2_O). (v) Subsequently, TMAH window etching and heater pad exposition were realized by reactive ion etching (Sentech SI 591). (vi) Further, the 10/200 nm Ti/Pt interdigital testing electrodes were deposited like the heater, and (vii) Pd/SnO_2_ film patterns were integrated into the central area by self-assembly. (viii) Finally, the 3D cantilever was released by 25 wt% TMAH wet etching for 5 h at 85 °C.

### Film and Chip Characterization

Scanning electron microscope–energy-dispersive spectroscopy (SEM–EDS, Gemini SEM 300) was utilized to characterize the morphology of materials, the composition of film, and the structure of sensors. Raman spectroscopy (LabRAM HR800) was used to recognize detachment of the surface SnO_2_ film, and the SnO_2_ crystal structure was characterized by X-ray diffraction (XRD, Thermo Scientific Talos F200X). Ellipsometer (RC2 XI) and atomic force microscopy (AFM, Bruker Dimension EDGE) were applied to evaluate the thickness and surface change of SiO_2_ and HfO_2_ films during wet etching. X-ray photoelectron spectroscopy (XPS, Thermo Scientific K-Alpha) was utilized to study the coverage of HfO_2_ on the SiO_2_ with different thicknesses. For 50c HfO_2_, transmission electron microscope (TEM, Thermo Scientific Talos F200X) was used to display the fine structure.

### Gas Sensing Measurement

During measurements, the sensors were placed in a fully sealed 20 L chamber. After preheating, their signals stabilized, and target gases were subsequently injected. Once the sensor reaction was completed, the chamber was opened to exhaust the gases until the signal returned to the baseline, after which the next test cycle was performed. Throughout the measurements, humidity and temperature were maintained at 40% ~ 45% RH and 22 ~ 23 °C. The sensors were connected in series with a reference resistor of known value R_reference_, and a fixed voltage V_fix_ was applied across the circuit. The sensor resistance R_sensors_ was then determined using the voltage divider principle (R_sensors_ = (V_fix_-V_reference_)/V_reference_ × R_reference_), where V_reference_ is the measured voltage across the reference resistor. A multimeter/DC power supply (U3606A) served both as the heating voltage source and as the signal acquisition unit, connected to the PCB wiring inside the sealed chamber. The response was defined as S = R_air_/R_gas_, where R_air_ and R_gas_ are the resistances R_sensors_ measured in air and in the target gas, respectively. The response time was defined as the time required for the sensitivity to change from its initial value of 1% to 90% of the total change. The operating temperature was calculated by averaging temperatures at four points along the isotherm, which was built through simulation. The relative maximum absolute deviation (RMD) and the relative standard deviation (RSD) of region of interest are calculated using five selected points: RMD = ΔT/mean × 100% and RSD = σT/mean × 100%.

### Micro-Hotplate Simulation

Micro-hotplate was simulated using finite element analysis method based on previous work [32, 33]. The device geometry was built first, and the device material was assigned to each domain [33]. Joule heating was implemented by adding a fixed voltage and ground to the heater electrodes. Heat loss mechanisms included conduction throughout the solid device and convective heat transfer to ambient air for all exposed external surfaces. The model employed a fine mesh with element sizes between 10 μm (minimum) and 80 μm (maximum). A stationary (steady-state) study was used to obtain the solution. The results such as the electric field of the heater or the thermal field distribution of the surface can be effectively obtained. The isotherm was constructed by uniformly dividing the temperature difference between the peak temperature T_peak_ and the room temperature T_room_ into 25 intervals. The boundary temperature T_iso_ of the isotherm corresponds to one interval below the peak temperature: T_iso_ = T_peak_—(T_peak_-T_room_)/25.

## Results and Discussion

### Self-Assembly and Silicon-Based Integration of Nanomaterials

**Fig. 2:**
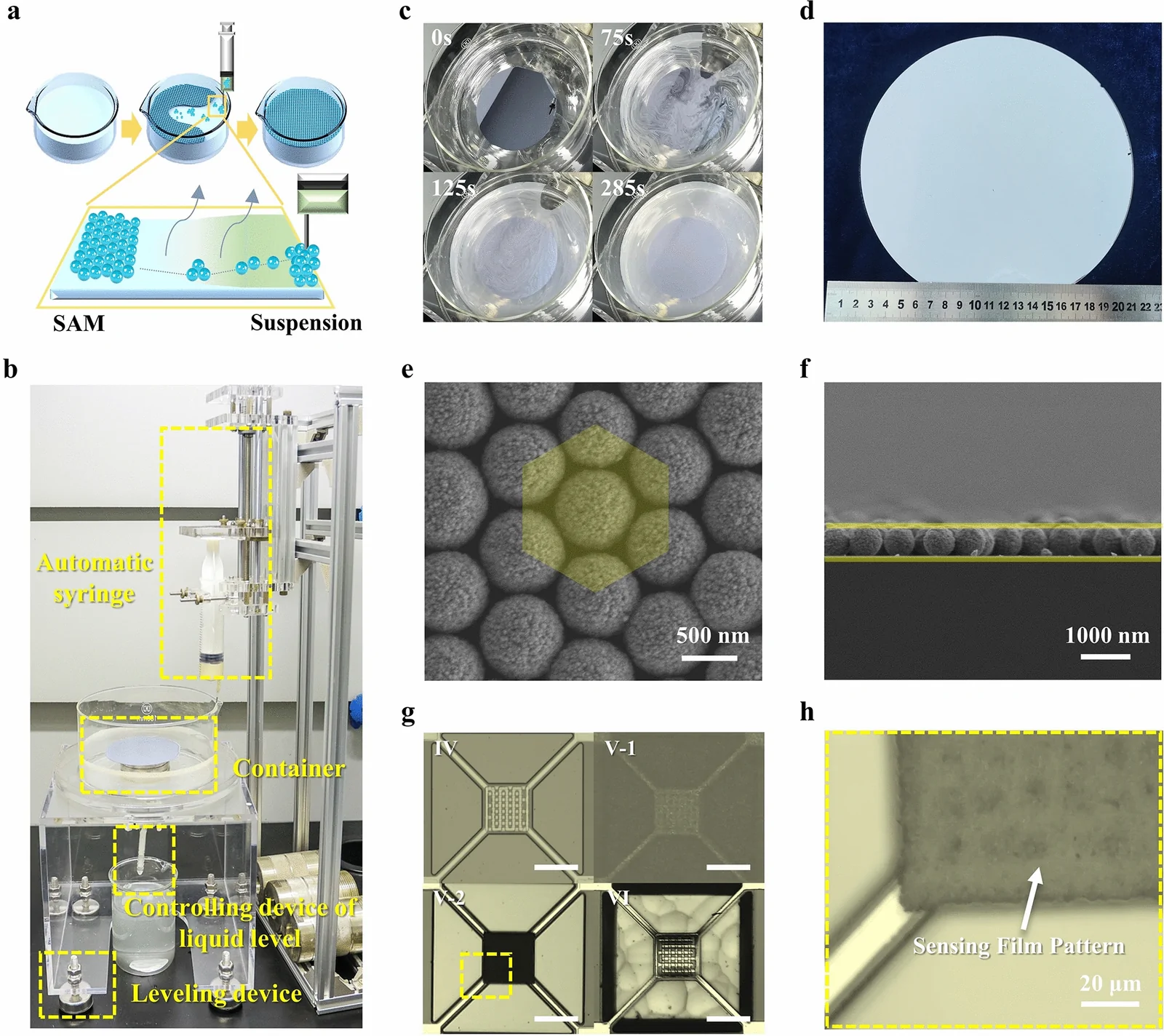
Self-assembly and integration of nanomaterials on 8-inch wafers. **a** Schematic diagram of the self-assembly process. **b** Photograph of the self-assembly equipment. **c** Photographs showing different stages of the self-assembly process. **d** Photograph of an 8-inch silicon wafer uniformly covered with a SnO_2_ SAM. **e–f** Top-view and Cross section SEM images of the SnO_2_ SAM. **g** Optical microscope images of MEMS gas sensing chips during fabrication. Scale bars are 100 μm. Corresponding to Fig. 1: (IV) preparation of heater and IDEs, (V-1) transfer of SnO_2_ SAM onto the surface, (V-2) patterning of the SAM via lift-off, and (VI) release of cantilever beams by TMAH wet etching, during which the sensing film was removed. **h** Enlarged optical microscope image of **g** (V-2), showing the directional integration of the sensing film

Large-area film formation is essential for achieving wafer-level integration of nanomaterials. Among various strategies, self-assembly stands out as an effective method across scales [34, 35], exploiting surface tension gradient of the suspension–water interface to drive material diffusion under the Marangoni effect (Fig. 2a). Driven by interparticle repulsion together with the combined effects of dispersants and surface tension, nanoparticles are confined to the interface, where they preferentially organize into a monolayer rather than multilayer stacks. During solvent evaporation, capillary forces further consolidate this arrangement into a tightly packed self-assembled monolayer (SAM), whose uniformity is crucial for consistent sensing performance by minimizing variations in film properties that could otherwise affect sensor responses.

For wafer-level SAM fabrication, maintaining a stable injection over extended periods is crucial. However, manual injection often leads to nanomaterial stacking or film fracture due to fatigue from prolonged fixed postures, thereby degrading film quality. In addition, once the film forms at the liquid–air interface, the manual transfer process makes it difficult to consistently control the angle and transfer speed. To address these challenges, a customized self-assembly setup (compatible with wafers up to 8 inches) was developed, as shown in Fig. 2b, capable of precisely controlling key parameters such as injection height and angle, injection rate, film transfer rate, and the contact angle between the wafer and the liquid surface.

The feasibility of this setup was demonstrated using SnO_2_ nanosphere as a representative gas sensing material. Through strictly control of processing parameters, SnO_2_ nanospheres with high size uniformity and dispersion were achieved to suppress material aggregation and the irregular cracks in the resulting SAM [36, 37]. For the following self-assembly process (Figs. 2c and S2), injection of the SnO_2_ ethanol suspension on the water surface gradually transforms the film from a loose network to a dense monolayer. The gap at the injection site progressively narrows until the coverage reaches 100%. The liquid level is then lowered to non-destructively transfer the intact film onto the wafer surface. The resulting film exhibits a uniform color across the wafer without visible damage, as shown in Fig. 2d. At higher magnification, the ~ 700 nm SnO_2_ nanospheres arrange into a dense, hexagonally packed SAM, following the principle of energy minimization (Fig. 2e, f) and providing stable electron transport pathways during sensing.

Notably, the self-assembly process occurs naturally in deionized water at room temperature. This mild approach ensures the pre-fabricated MEMS device structure remains fully preserved during film formation. Crucially, because the synthesis and annealing steps are completed prior to self-assembly, this decoupled strategy provides the flexibility to easily modulate the nanomaterial structure and fully utilize its intrinsic excellent sensing performance. This enables large-area films of diverse advanced functional nanomaterials to be integrated using the same approach.

Building on this capability, a wafer-level process flow was proposed that transfers nanomaterials prior to wet etching. This “film-first, cantilever-later” strategy (Fig. S3) patterns the film on a flat structure, enabling arbitrary definition of the sensing area and avoiding cantilever damage during photolithography. Consequently, it effectively addresses the challenge of preparing films onto designated areas of suspend three-dimensional structures in conventional processes. Following this approach, the photoresist spin speed was reduced to increase its thickness, yielding a step height > 1.633 μm. This enabled a clean lift-off of the SnO_2_ nanospheres without multilayer edges [38], as shown in Fig. S4. After transferring SnO_2_ to the central sensing area (Fig. 2g, h), however, a major issue emerged during the final TMAH wet etch, namely, complete loss of the sensing film.

### HfO_2_ Interface Passivation Patterning Technology

**Fig. 3:**
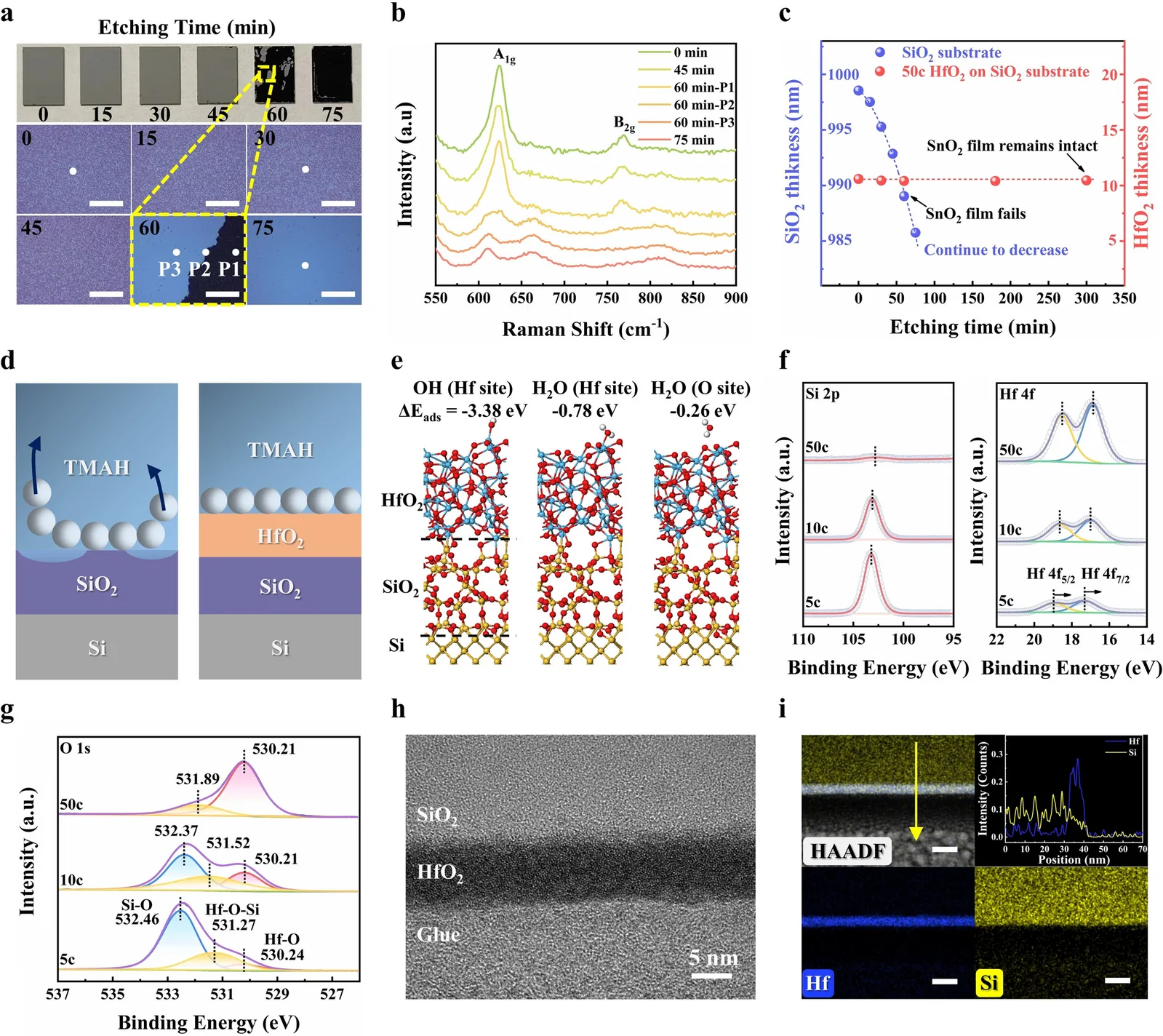
Failure mechanism analysis of SnO_2_ films and protective effect of HfO_2_ passivation. **a** Photographs and optical microscope images of SnO_2_ films deposited on SiO_2_ substrates after different etching durations. Enlarged optical images highlight surface changes corresponding to each etching time. Scale bars are 500 μm. **b** Raman spectra of the etched SnO_2_ films. For the 60-min sample, three spectra represent measurements at distinct positions marked by white dots in **a**. **c** Ellipsometer results showing thickness variations in SiO_2_ and 50c HfO_2_ films as a function of etching time. Dotted lines indicate fitted trends. **d** Schematic diagram of the completely differnet etching process of SnO_2_ films on the SiO_2_ and HfO_2_ substrates. Grey spheres represent SnO_2_ nanospheres. **e** Adsorption energy calculation results of an amorphous HfO_2_/SiO_2_/Si structure model. **f-g** High-resolution XPS spectra of 5c, 10c, and 50c HfO_2_ films showing the Si 2*p*, Hf 4*f*, and O 1*s* regions. **h** HRTEM image of 50c HfO_2_ films prepared on SiO_2_ substrates. **i** Elemental mapping of HRTEM image shown in **h**. The top-right panel presents the distribution of Hf and Si elements along the yellow arrow in the top-left image. Scale bars are 5 nm

To investigate the cause of film failure, SnO_2_ nanospheres were primarily self-assembled on the SiO_2_ substrate (as shown in Fig. S5). A quasi-in situ analysis was then conducted for SnO_2_ SAMs subjected to different etching durations. As shown in Fig. 3a, the film remains intact throughout the early stages, with a sudden, dramatic change appearing only after 60 min of etching—rather than peeling or cracking progressively from the start. Raman spectra (Fig. 3b) reveal that the P1 position on the 60 min sample retains peak positions and intensities identical to those at 0, 15, 30, and 45 min samples, corresponding to the expansion (A_1g_) and contraction (B_2g_) vibration mode of Sn–O bonds [39]. By contrast, the P3 position on the same 60 min sample shows spectra identical to the 75 min sample, featuring only SiO_2_/Si background peaks instead of SnO_2_. This sharp contrast within a single sample suggests that direct dissolution of SnO_2_ by TMAH is not the main cause of film failure. Supporting this, similar failure is observed for SnO_2_ films deposited on Si_3_N_4_ substrates after long etching durations (Figs. S6 and S7).

The SnO_2_ SAM was then removed from the SiO_2_ surface for further analysis. Measurements reveal a slight reduction in SiO_2_ thickness (from 998.55 to 985.76 nm, as shown in Fig. 3c), indicating that the contact interface between the SiO_2_ insulating layer and the SnO_2_ sensing layer is destroyed. This suggests that TMAH solution diffuses through the porous SnO_2_ film [40] and attacks the underlying SiO_2_ layer. Once the sole anchoring point between film and substrate is broken, the film readily separate from the surface (Fig. 3d). To clarify the SiO_2_ etching mechanism and develop a solving strategy, the chemical reaction energy (ΔE_ads_) of an alkaline aqueous solution to amorphous SiO_2_ was calculated (Fig. S8). The low adsorption energy (− 3.81 eV) means that OH species readily adsorbs and reacts on the surface of SiO_2_. Etching proceeds via the formation of soluble coordination complexes between OH species and Si atoms [41], much like in crystalline silicon. However, in SiO_2_, the highly charged Si ions are partially shielded by polarizable O ions, requiring time for TMAH to overcome this shielding [42, 43]. This slows the apparent etching rate, making SiO_2_ etching behavior easy to overlook compared to Si. In this case, however, even this mild etching must be treated as a critical concern.

A common way to mitigate such interface degradation is to insert a functional interlayer between substrate and top film [44, 45]. Following this principle, it is expected to add an interface passivation layer to block the Si exposure and further protect SnO_2_ film. HfO_2_ is a common high-k dielectric material extensively utilized in advanced electronic devices [46–48]. It offers higher density and greater thermodynamic stability than SiO_2_ [49]. These superior properties expected to be helpful in resisting long-term etching in alkaline solutions.

Before preparing interface passivation layer, the chemical reaction energy of the alkaline aqueous solution on amorphous HfO_2_ was first calculated (Fig. 3e). Higher adsorption energy of OH species on HfO_2_ indicates lower reactivity toward alkaline solutions compared to SiO_2_. Conversely, H_2_O has even lower adsorption energy on HfO_2_ than SiO_2_, occupying potential reaction sites and further reducing OH species participating probability in the etching reaction. This effect becomes more pronounced in crystalline HfO_2_ (Fig. S8). These results suggest that HfO_2_ is an ideal candidate for potential interface passivation.

HfO_2_ interface passivation layer was prepared via atomic layer deposition (ALD), which allows precise tuning of thickness and surface chemical composition through operation mode and parameters controlling [50]. According to XPS analysis (Fig. 3f, g), low ALD cycles results in the formation of Hf silicates at the interface, still with strong Si–O contributions (the peak in O 1* s* region originates from Si–O, Hf–O-Si, and Hf–O bonds) [51]. Increasing the cycle count gradually transforms the interface chemistry from Hf silicate to HfO_2_, shifting peaks to lower binding energy (BE) [52]. Meanwhile, the increased electron donor Hf induces higher electron density around Si atom, enhancing the screening of metal core levels and leading to a lower BE in Si 2*p* region [53]. After 50 cycles, the higher ratio of Hf–O to Si–O in O 1*s* suggests that the underlying SiO_2_ is almost fully shielded, with the Si 2*p* peak nearly vanishing.

A 50-cycle HfO_2_ passivation layer was therefore selected for validation. TEM image (Fig. 3h) reveals a uniform amorphous HfO_2_ film (~ 10.5 nm) fully covering the SiO_2_ surface. Elemental analysis (Fig. 3i) further confirms complete coverage, as the Si signal decreases while the Hf signal increases from bulk to surface. Even after 5 h of TMAH etching, the HfO_2_ layer retains its thickness with negligible change (Fig. 3c), demonstrating outstanding anti-etching stability. Building on these results, a protection strategy was proposed: inserting an HfO_2_ interlayer between the SiO_2_ insulating layer and the SnO_2_ sensing layer to provide effective passivation (Fig. 3d).

**Fig. 4:**
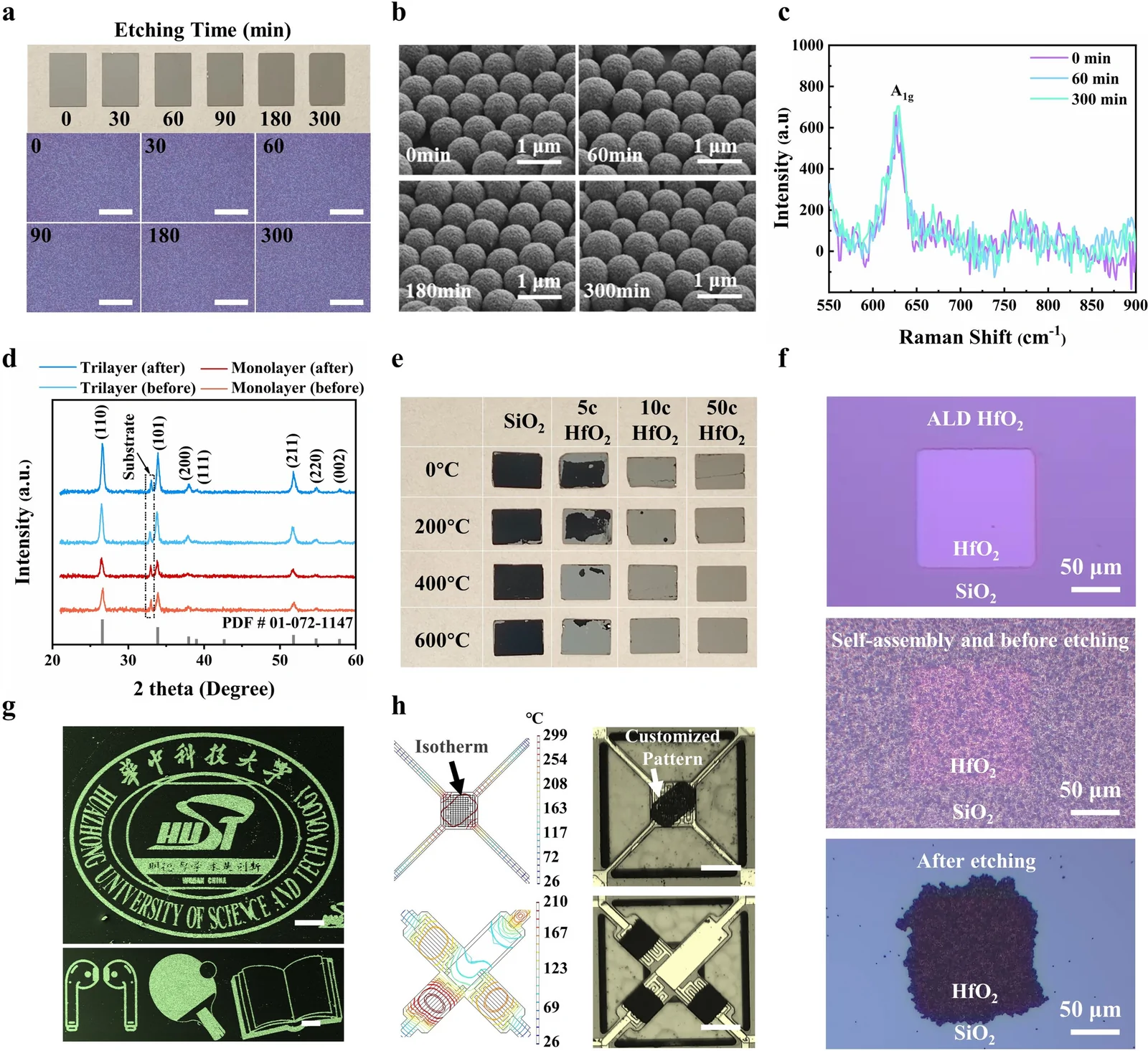
Protective performance of HfO_2_ interface passivation patterning technology. **a** Photographs and optical microscope images of SnO_2_ films on HfO_2_ substrates after different etching durations. Scale bars are 500 μm. **b** SEM images of SnO_2_ nanospheres after etching. **c** Raman spectra of SnO_2_ films etched for varying durations. **d** XRD patterns of monolayer and triplayer SnO_2_ films before and after wet etching. **e** Photographs of SnO_2_ film with different HfO_2_ passivation layers thicknesses and annealing temperatures after 5 h etching. **f** Optical microscope images of SnO_2_ films on both SiO_2_ and HfO_2_ substrates subjected to simultaneous etching. **g** SEM images of various SnO_2_ film patterns after etching. Scale bars are 200 μm. **h** Thermal distribution (simulation results) of two different types of MEMS gas sensing chips and the corresponding customized SnO_2_ film patterns (optical images of the devices, scale bar 100 μm)

Gas sensing performance is closely tied to the morphology and crystallinity of sensing film. To evaluate these aspects, self-assembled SnO_2_ films with and without TMAH etching were compared (Figs. S9 and 4a). As depicted in Fig. 4a, b, even after prolonged etching for up to 300 min, the SnO_2_ film remains intact, preserving its ordered porous nanosphere structure. Meanwhile, Raman spectrum (Fig. 4c) and XRD patterns (Fig. 4d) retain the characteristic SnO_2_ peaks without noticeable shift or intensity loss, confirming that the crystallinity is well maintained. These results demonstrate that the SnO_2_ film protected by HfO_2_ withstands long-term TMAH etching without structural or crystalline degradation.

All the above HfO_2_ films were annealed in air after ALD to avoid oxygen-deficient HfO_x_, which could otherwise reduce SiO_2_, causing SiO_x_ diffusion along grain boundaries [49] or generating HfO_2_ stoichiometric defects [54]. Annealing temperature is also critical. According to the results in Fig. 4e, 200 °C represents the minimum temperature required to achieve reliable anti-etching performance, whereas excessive heating risks damaging pre-fabricated structures, such as warping and falling off from metal film at 800 °C (Fig. S10). The optimal process window is therefore 200 ~ 600 °C, providing sufficient tolerance for both upstream and downstream fabrication processes. Thickness is equally important. Figure 4e indicates that films thinner than 10.5 nm lose effectiveness, consistent with XPS observations (Fig. 3f, g). A direct comparison between SiO_2_ and HfO_2_ regions after etching (Fig. 4f) reveals dramatically different outcomes. SnO_2_ survives exclusively on the rectangular HfO_2_ areas. Minor edge irregularities arise from SnO_2_ film bridging over the pattern edges, indicating that fine patterns still require photolithographic definition.

This strategy also demonstrates capabilities in film thickness control, precise patterning, and compatibility with diverse materials. SnO_2_ films with different thicknesses were prepared via layer-by-layer self-assembly [55], and trilayer films retain crystallinity after etching (Fig. 4d). Patterns matched to standard MEMS hotplate geometries were fabricated (Fig. S11) [7, 8, 24, 29, 56–58], and arbitrary patterns were also achievable using maskless direct writing lithography (Fig. 4g), though line widths below 10 µm exhibited blurred edges—likely limited by lithographic resolution and nanosphere size, not the HfO_2_ passivation. Furthermore, temperature plays a crucial role in governing gas adsorption and surface reactions on sensing materials [59], making uniform working conditions essential for reliable mechanism studies. Simulations (Fig. S12) reveal that isothermal zones are typically irregular rather than ideal geometric shapes [32, 33]. Patterning sensing films within this isothermal-defined range of interest (Fig. 4h) rather than using the conventional rectangular area (Fig. S13), substantially improves the thermal uniformity—the relative maximum absolute deviation decreases from 36.89% to 8.60%, and the relative standard deviation decreases from 14.28% to 3.23%. This demonstrates the methodological significance of the proposed patterning strategy for fundamental sensing research.

Except for SnO_2_, this passivation approach also benefits other sensing nanomaterials, provided they are intrinsically stable in TMAH. For example, TMAH-resistant In_2_O_3_ nanosphere films remain intact after prolonged etching (Figs. S14 and S15). Conversely, materials like ZnO dissolve and are thus unsuitable (Fig. S16).

### High-Performance 3D MEMS Gas Sensing Chips

This protection strategy has been successfully applied to fabricate sensing films with tunable thickness, achieve arbitrary pattern transfer, and assist film to resist long-term wet etching on two-dimensional surfaces. Ideally, it can be extended to wafer-scale manufacturing of 3D MEMS sensing chips while remaining compatible with advanced functional nanomaterials.

H_2_ is increasingly used across energy and industrial sectors. Its wide flammability range and colorless and odorless properties make reliable detection essential. Metal oxide semiconductor-based H_2_ sensing chips are particularly attractive due to their small footprint, fully solid state, and low power consumption. Substantial progress has been achieved through structure modification [60, 61] (e.g., vacancies, porous), catalytic decoration [62–64] (e.g., Pd, Pt, Au), and heterojunction design [65, 66].

**Fig. 5:**
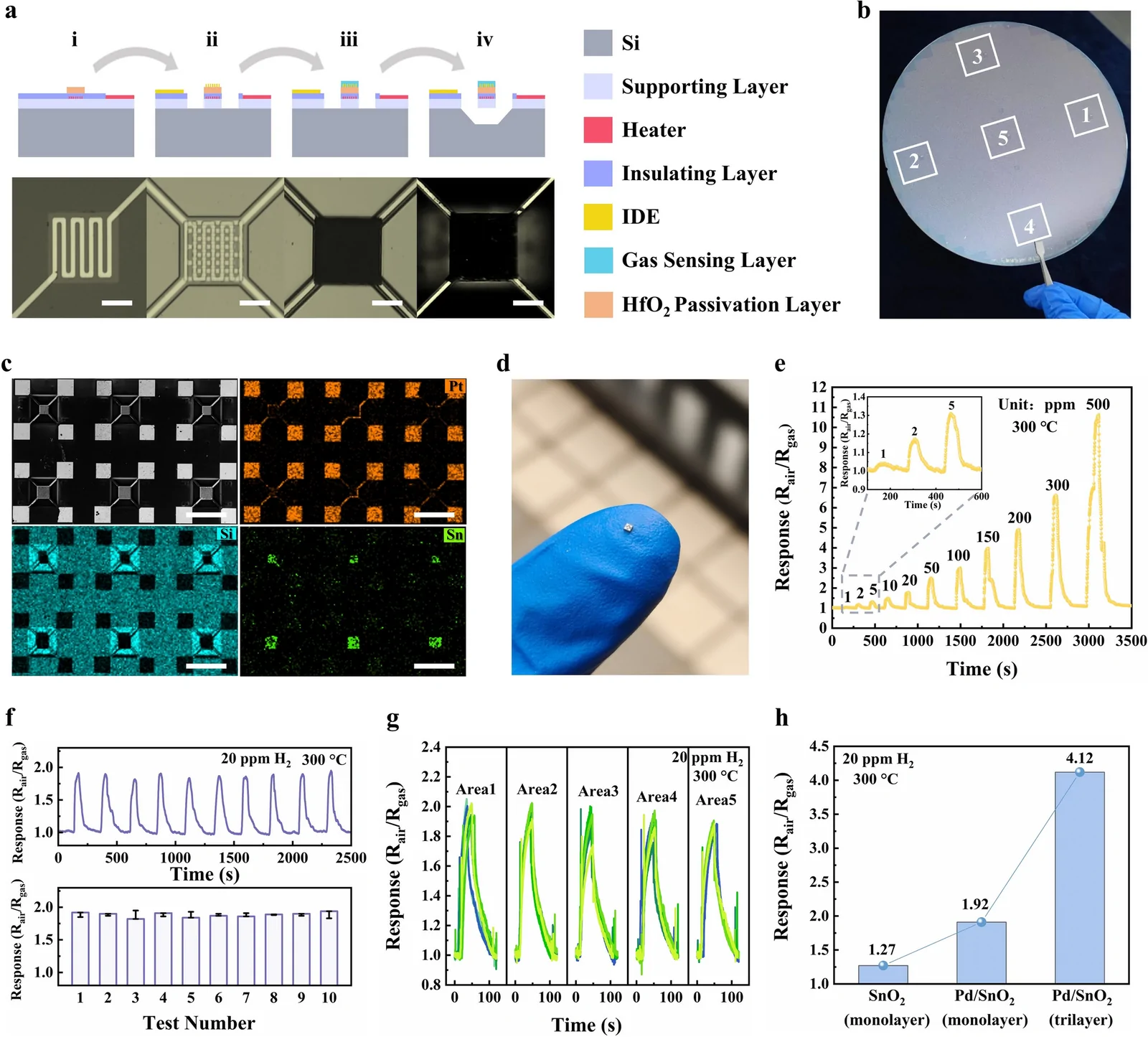
Fabrication process and performance evaluation of MEMS H_2_ sensing chips. **a** Schematic illustration of the fabrication process, highlighting the additional steps introduced by the HfO_2_ passivation layer, along with corresponding optical images of the core MEMS structure. Scale bars are 50 μm. **b** Photograph of an 8-inch wafer containing completed Pd/SnO_2_ H_2_ sensing chips. **c** SEM–EDS images of the fabricated sensing chips before dicing. Scale bars are 500 μm. **d** Photograph of an individual Pd/SnO_2_ H_2_ sensing chip after dicing. **e** Sensing responses of a monolayer Pd/SnO_2_ sensor exposed to H_2_ concentrations from 1 to 500 ppm at 300 °C (RSD = 3.23%).** f** Repeatability test of a monolayer Pd/SnO_2_ sensor over 10 consecutive exposures to 20 ppm H_2_. Error bars indicate the absolute deviation from the mean response. **g** Response variation of 25 monolayer Pd/SnO_2_ sensors randomly selected from five different regions of the wafer. **h** Response comparison of various sensors loading SnO_2_, monolayer Pd/SnO_2_, and trilayer Pd/SnO_2_ sensing films

Among these approaches, Pd decoration has proved especially effective because Pd nanoparticles promote dissociative adsorption of H_2_ and accelerate spill-over processes on sensing materials surfaces [26]. Inspired by these advantages, Pd-decorated SnO_2_ (Pd/SnO_2_) nanospheres were therefore integrated into an 8-inch wafer under the newly established process flow (Figs. 5a, b, and S17), and an HfO_2_ passivation layer was introduced between the insulation layer and the testing electrode. Figure 5a shows the evolution of the core MEMS structure through key steps including ALD HfO_2_, IDE deposition, Pd/SnO_2_ self-assembly, and TMAH wet etching. The final sensing chips (Fig. 5c) retain their structural integrity after etching, with the sensing film confined to the central region and no contamination of peripheral areas. The diced dies (Fig. 5d) highlight the compact footprint (900 × 900 × 500 μm^3^) enabled by MEMS process, demonstrating that HfO_2_ interface passivation patterning technology truly supports wafer-level manufacturing of tens of thousands of 3D MEMS gas sensing chips compatible with advanced functional nanomaterials.

The fabricated chip-based gas sensors exhibit optimal sensitivity to H_2_ at 300 °C (Fig. S18) and clearly distinguish concentrations from 1 to 500 ppm (Fig. 5e). Within this wide sensing range, a proportional, linear relationship between response and concentration (Fig. S19) indicates that unknown concentrations can be quantified with simple calibration. Repeatability testing with 20 ppm H_2_ injection over 10 cycles (Fig. 5f) demonstrates a low relative standard deviation of 1.97%, confirming excellent repeatability. Compared with previous studies (Table S1), the Pd/SnO_2_ nanospheres sensors reported here demonstrate superior manufacturing capability in both wafer-scale fabrication and film patterning. Actually, the performance of nanomaterials can be further optimized to fully exploit its potential. In comparison with the same wafer-level process [26], the present sensors achieve higher sensitivity (1.92 vs. 1.52) and markedly faster response (19.68 vs. 115.4 s), as shown in Fig. S20.

Consistency is a critical metric for gas sensors. According to Fig. 5g, 25 monolayer Pd/SnO_2_ sensors selected from five regions show similar dynamic responses to H_2_. A statistical distribution analysis (Fig. S21) reveals the best uniformity in Area5, whereas edge regions exhibit slightly weaker consistency. This variation reflects positional effects during fabrication, but industrial-scale equipment could further improve outcomes. Additionally, the high yield is evidenced by an RSD of 3.84% across all 25 sensors, well below the 5% benchmark, confirming excellent consistency. Importantly, the sensing film maintained its high intrinsic performance after wet etching.

This manufacturing process not only enables the intrinsic performance of nanomaterials to be improved via modification or doping prior to self-assembly, but also allows further optimization through regulating the number of self-assembled layers. For instance, a trilayer Pd/SnO_2_ sensing film fabricated via three self-assembly cycles exhibited significantly higher sensitivity than a monolayer (Figs. 5h and S22), owing to the combined effects of gas diffusion pathways [67] and electron conduction networks [68]. Furthermore, in view of the strong sensitivity of metal oxide to humidity [69, 70] and various environmental fluctuations, future work may leverage this wafer-level manufacturing process to integrate more types of sensing materials, thereby improving real-environment adaptability.

In summary, the proposed process enables scalable fabrication of MEMS sensors that preserve the intrinsic sensing properties of nanomaterials. Moreover, further enhancing sensing performance through modifications, doping, structural adjustments, or thickness control of the nanomaterials is readily achievable within the same manufacturing flow.

## Conclusions

This work introduces a wafer-level manufacturing strategy for MEMS sensing chips that enables the seamless integration of advanced functional nanomaterials, supported by a robust theoretical and process framework to ensure reproducibility and scalability.

Specifically, high-quality 8-inch SnO_2_ self-assembled monolayer films with hexagonal packing are non-destructively fabricated through a self-built setup. The uniformity of nanomaterials size and optimized concentration of suspension are identified as key preconditions for success. To address the intrinsic vulnerability of sensing films during wet etching, an ALD HfO_2_ passivation layer (~ 10.5 nm) is developed as an effective protection strategy, eliminating the soluble coordination between OH species and Si atoms and thereby preventing root failure of the sensing film in during the wet etching process. This interface passivation approach proves versatile, enabling the preparation of sensing films with various TMAH-resistant materials, pattern geometries, and thicknesses. Building on these advances, a new process flow is established for the wafer-scale manufacturing of Pd/SnO_2_ H_2_ sensing chips on 8-inch wafers. The resulting packaged sensors exhibit high sensitivity, fast response, and excellent consistency.

In summary, this wafer-level approach resolves the long-standing challenges of traditional transfer processes—low efficiency, excessive coverage, and thickness non-uniformity—thereby enabling the seamless integration of high-performance TMAH-resistant nanomaterials with suspended MEMS structures. This advance paves the way for scalable fabrication of high-performance MEMS sensing chips with enriched material diversity and enhanced functionality.

## Supplementary Information

Below is the link to the electronic supplementary material.

Supplementary file1 (DOCX 18763 kb)
