# Supplementary material for: Wafer-Level Self-Assembly and Interface Passivation Patterning Technology for Nanomaterial-Compatible 3D MEMS Sensing Chips
Source: Nanomicro Lett. 2026 Jan 26;18:221. doi: 10.1007/s40820-026-02080-4 (PMC12834869; doi:10.1007/s40820-026-02080-4)
Supplement: Supplementary file 1 — Supplementary file1 (DOCX 18763 kb) [file 40820_2026_2080_MOESM1_ESM.docx]

Supporting Information for

**Wafer-Level Self-Assembly and Interface Passivation Patterning Technology for Nanomaterial-Compatible 3D MEMS Sensing Chips**

Zheng Zhang^1^, Yanlin Zhang^1^, Yuanyuan Luo^2^, Guoliang Lv^1^, Jianglin Yin^1^, Pengwei Tan^1^, and Guotao Duan^1, 3, *^

^1^School of Integrated Circuits, Huazhong University of Science and Technology, Wuhan 430074, P. R. China

^2^Key Laboratory of Materials Physics, Institute of Solid State Physics, HFIPS, Chinese Academy of Sciences, Hefei 230031, P. R. China

^3^Wuhan National Laboratory for Optoelectronics, Huazhong University of Science and Technology, Wuhan 430074, P. R. China

*Corresponding author. E-mail: [duangt@hust.edu.cn](mailto:duangt@hust.edu.cn) (Guotao Duan)

**Supplementary Figures and Tables**


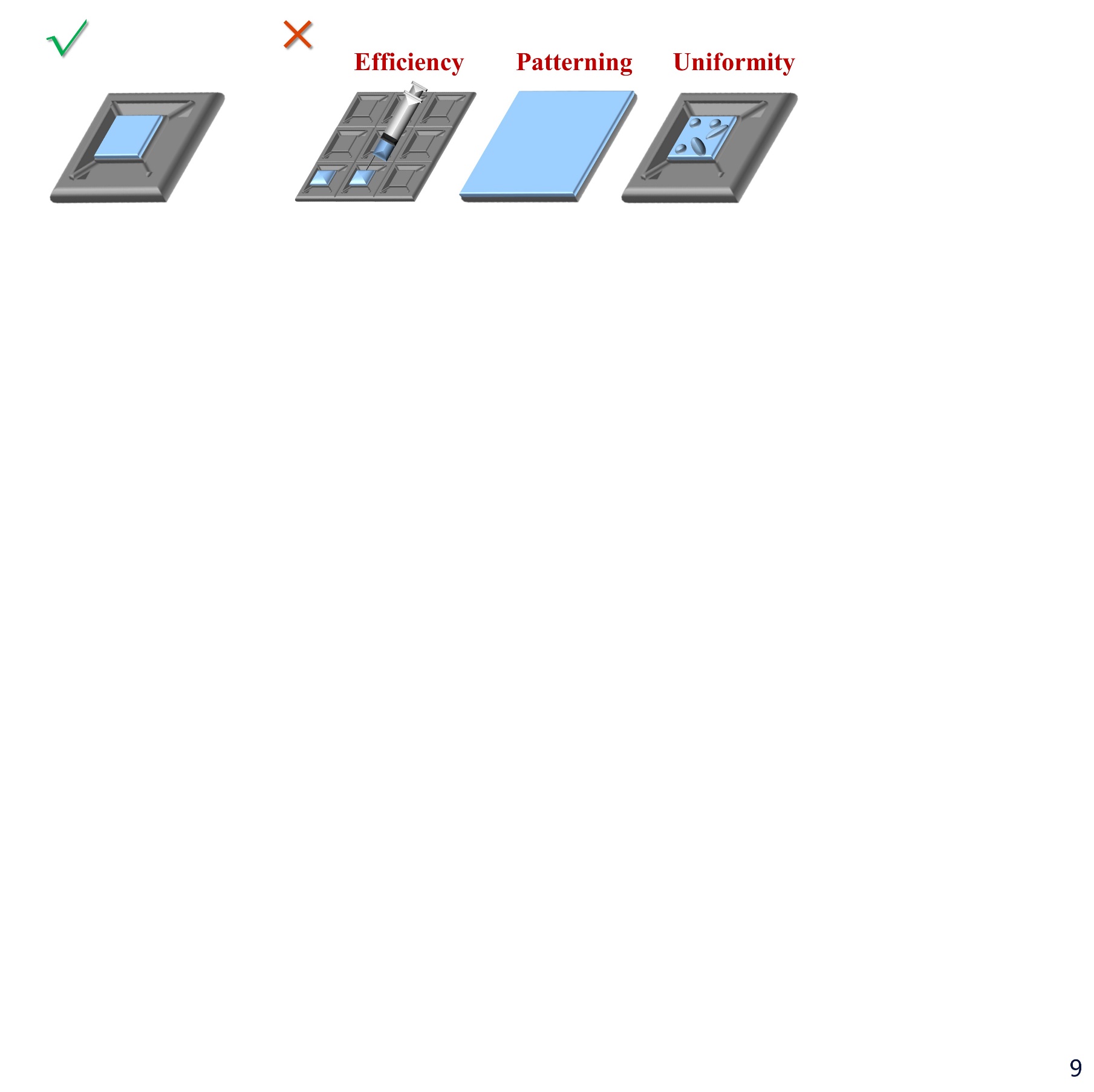


1. Schematic diagrams illustrating the expected effects and current challenges in nanomaterials film transfer. Challenges include improve transfer efficiency, thickness uniformity and accurately pattern transfer


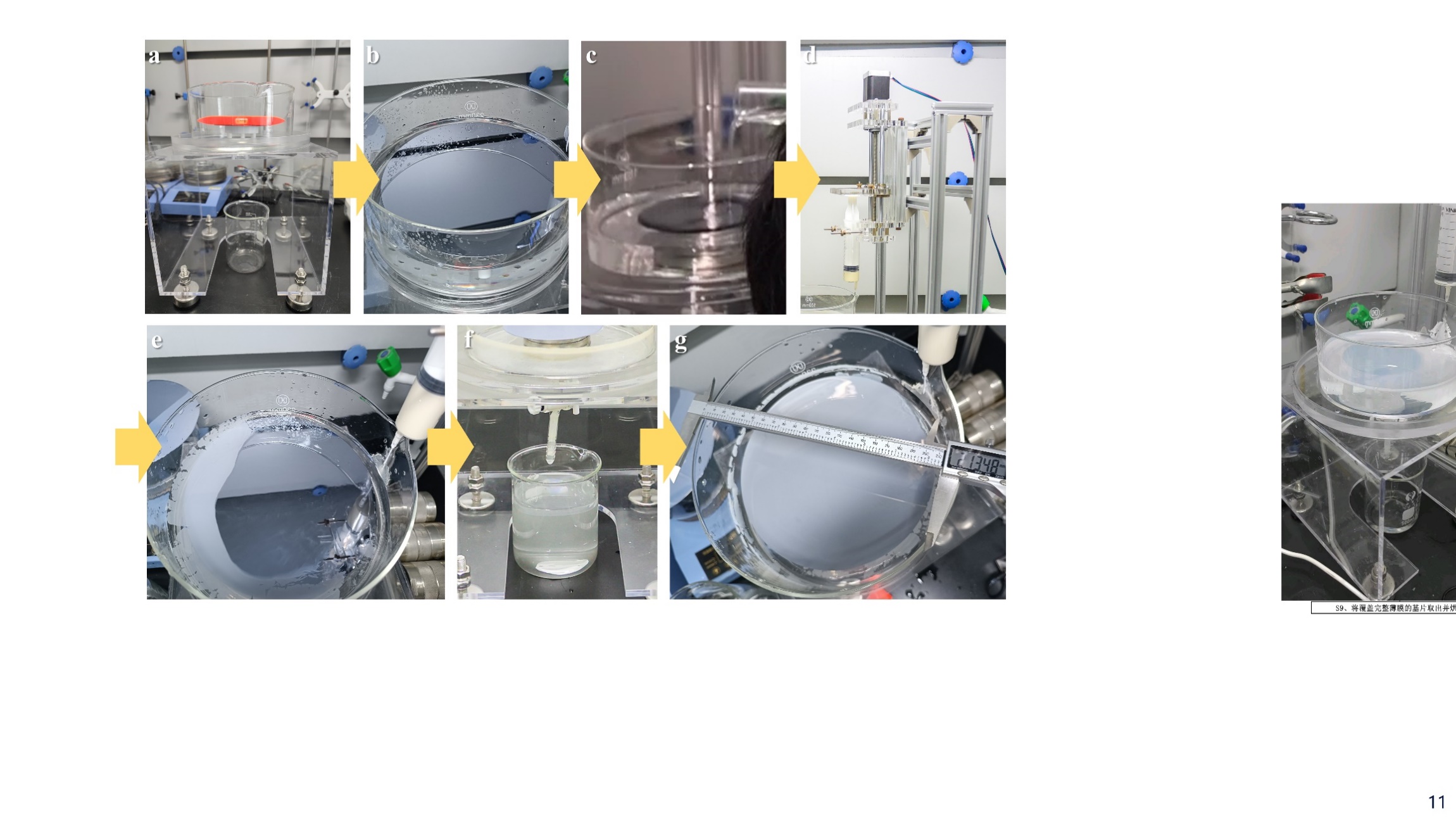


1. Operation process of the self-assembly equipment. **a** Container is adjusted horizontally by the bottom leveling device. **b** Target wafer is placed in the container. **c** DI water is added to submerge the entire wafer, with the liquid level 1 cm above the wafer surface. **d** Automatic syringe is assembled. **e** Automatic syringe is activated to inject suspension, and the films gradually form on the water surface as shown in Fig. 2c. **f** Liquid level controlling device is turned on to decrease the liquid level. **g** Self-assembly monolayer films are transferred onto the wafer surface as the liquid level decreases


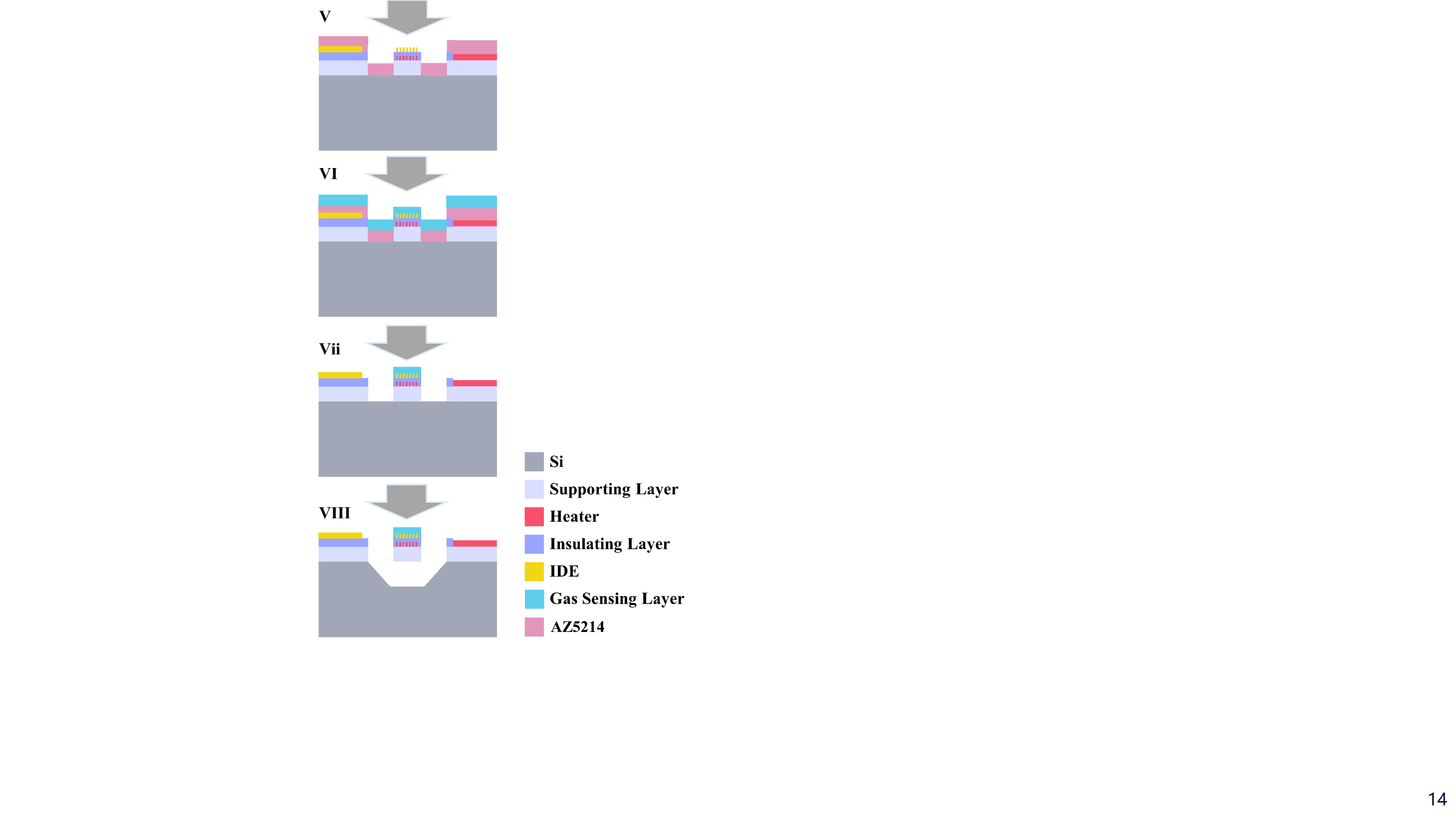


1. Schematic diagrams of the “film first, cantilever later” wafer-level process flow. Following the IDE deposition step as shown in Fig. 1, the subsequent steps are: (V) The film pattern are defined by lithography. (VI) Self-assembly is performed to form the sensing film. (VII) Unwanted film areas are removed by a lift-off process. (VIII) Cantilever stucture is released via TMAH wet etching


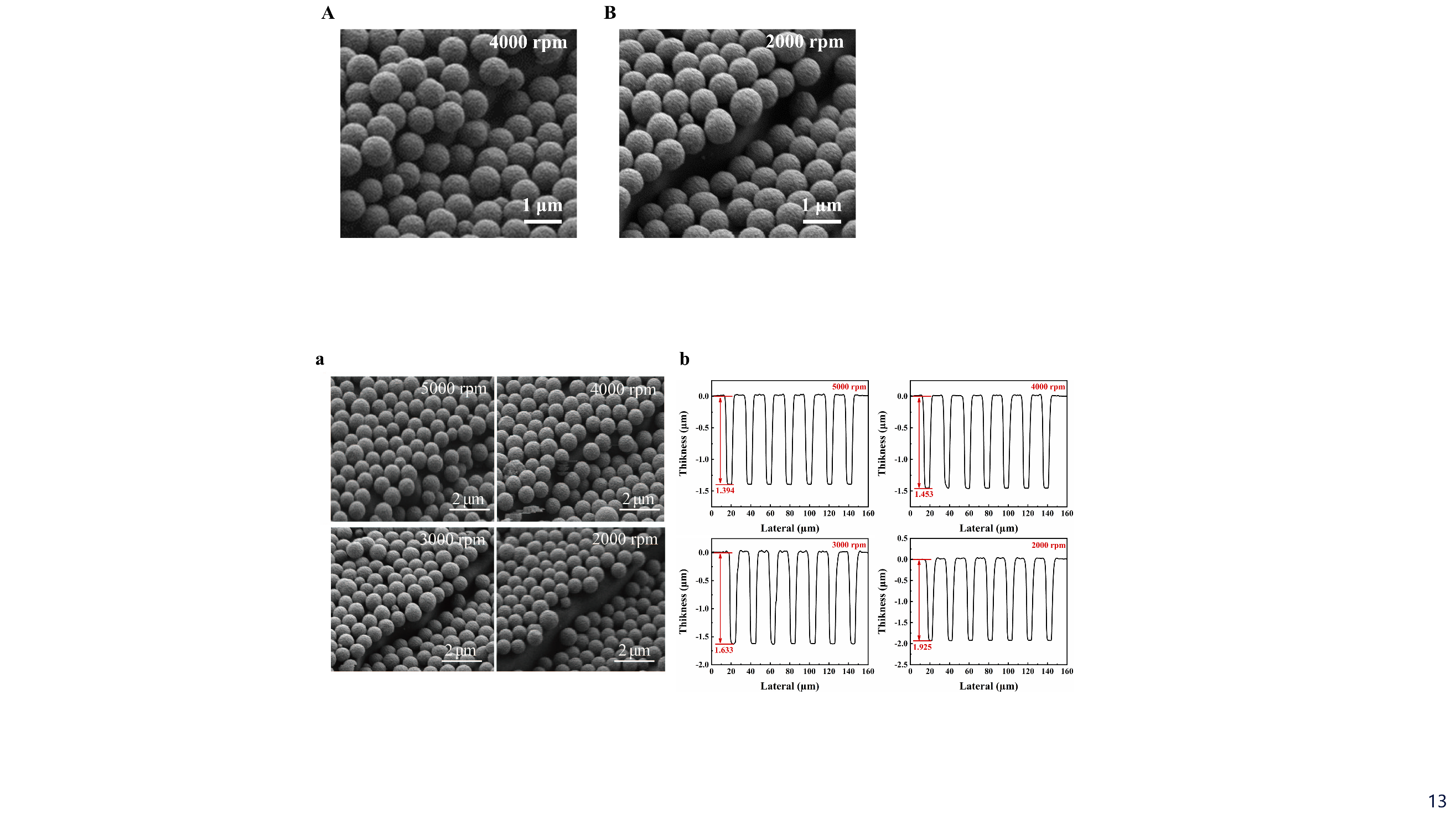


1. Self-assembly films prepared on photoresist layers of varying thickness. **a** SEM images showing the film seperation status. **b** Thicknesses of photoresist under different spin rates


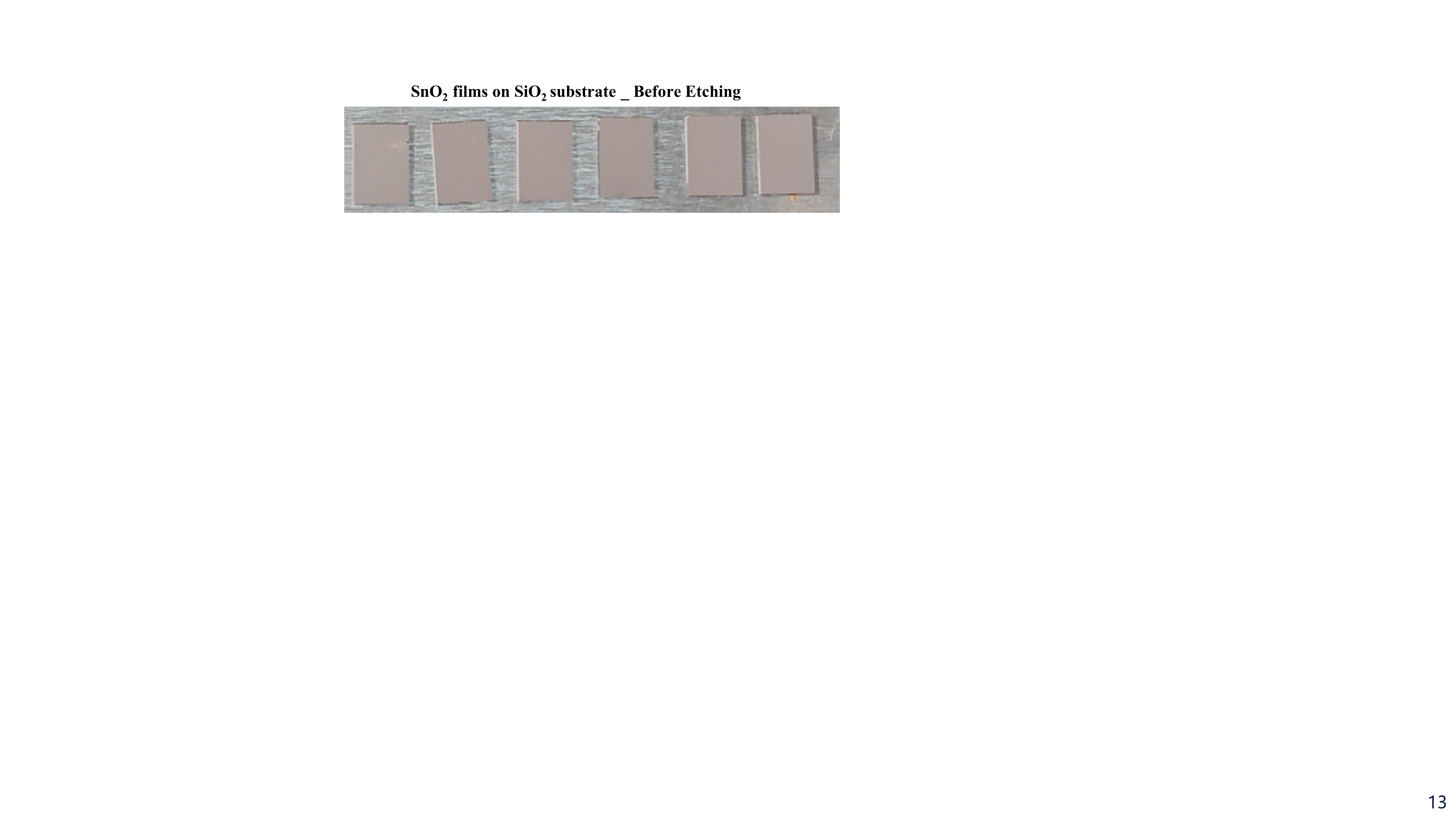


1. Photograph of the self-assembly monolayer SnO_2_ films before wet etching, deposited on a SiO_2_ substrate


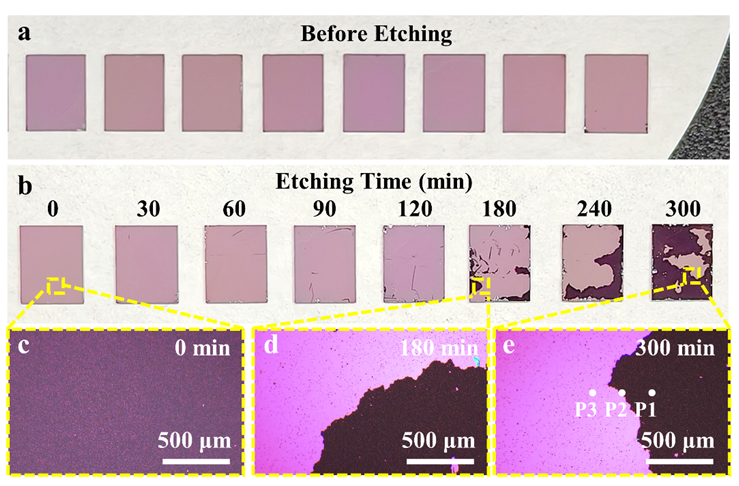


**Fig. S6 a-b** Photographs and optical microscope images of SnO_2_ films on Si_3_N_4_ substrates before etching and after different etching durations. **c-e** Enlarged optical images highlighting surface changes corresponding to each etching time


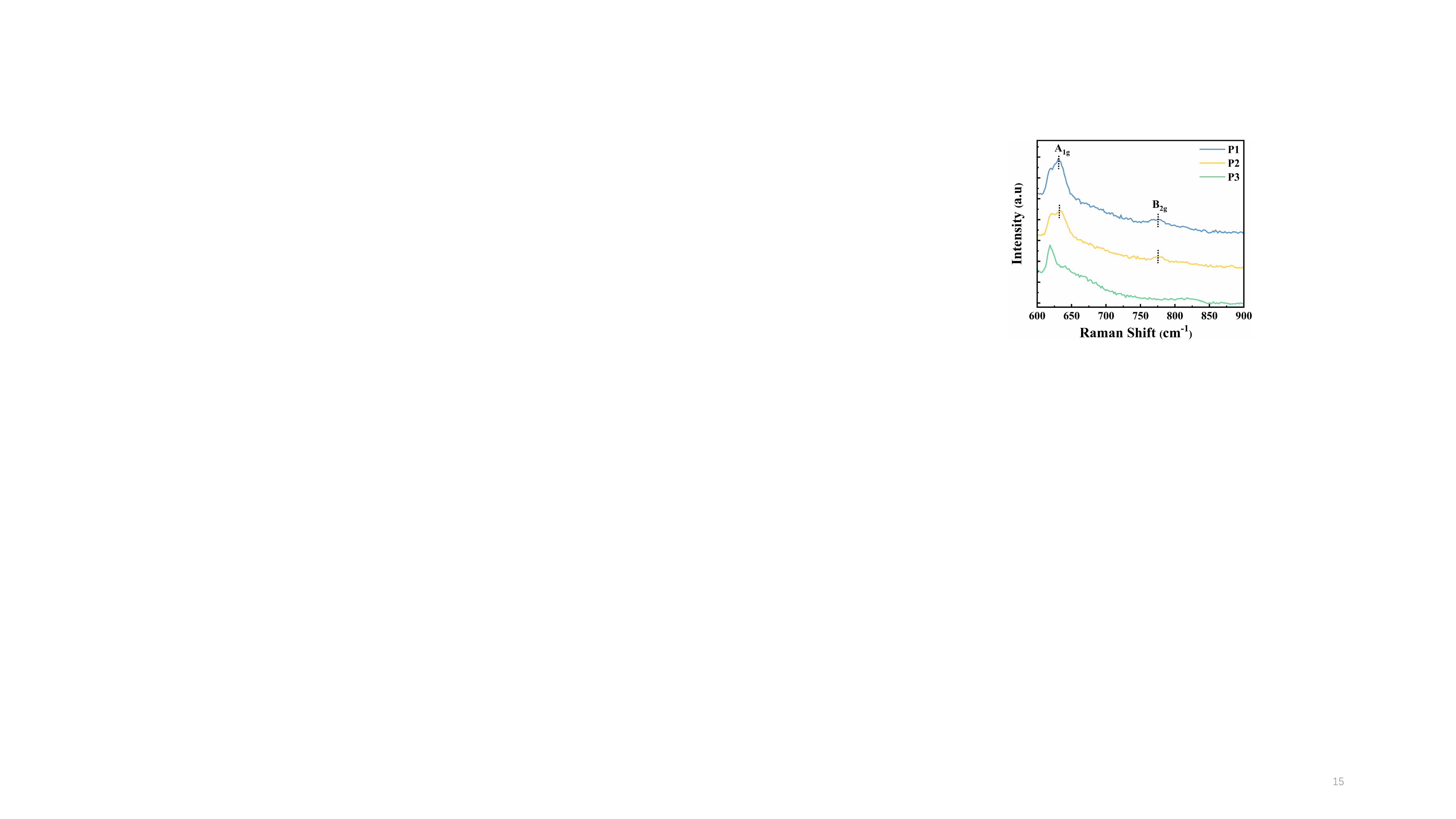


**Fig. S7** Raman spectra of the etched SnO_2_ films on Si_3_N_4_ substrates. For the 300-min sample, three spectra represent measurements at distinct positions marked by white dots in Fig. S9e. The spectra of P3 shows the absence of the classic SnO_2_ peak


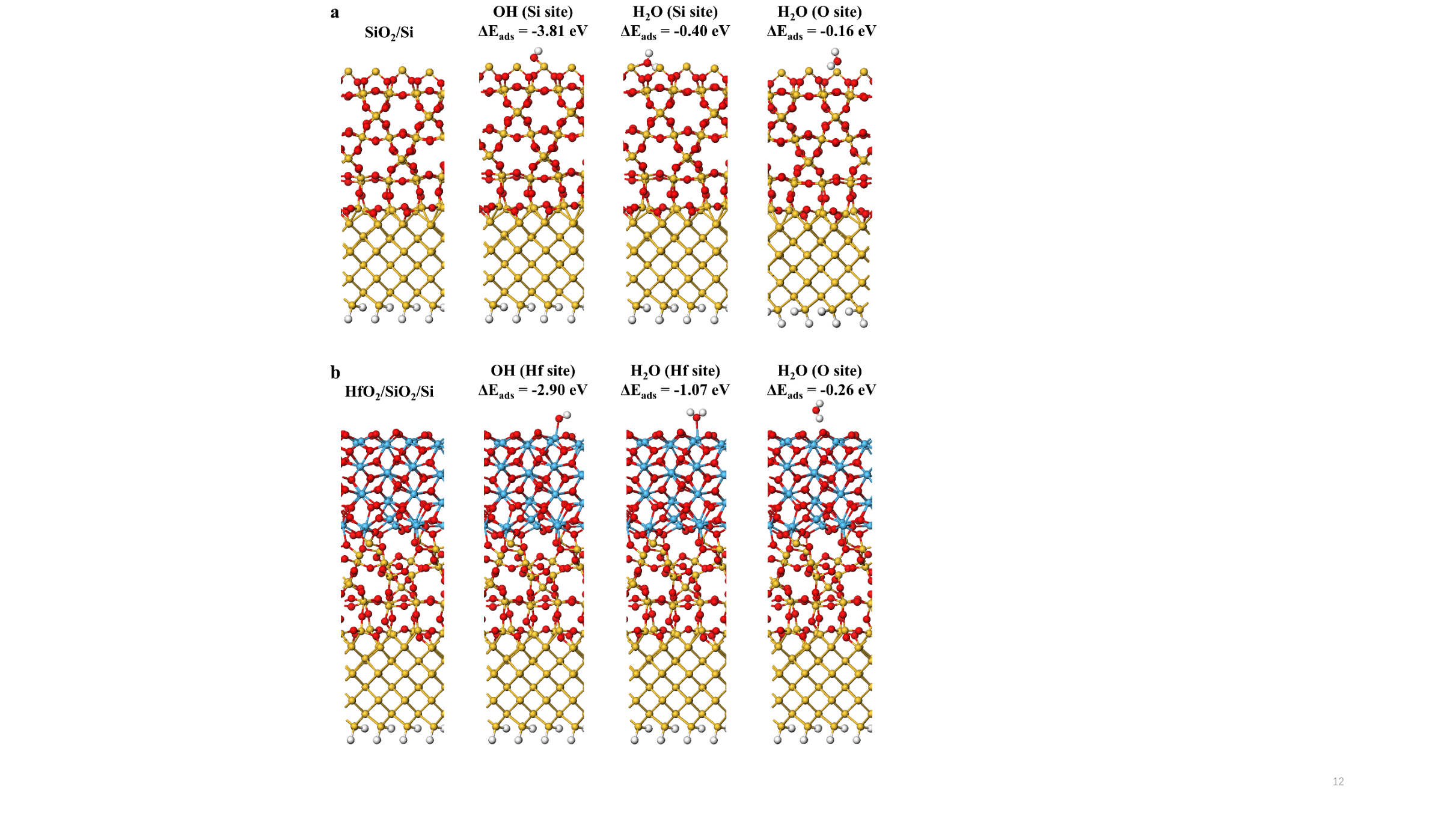


**Fig. S8** Adsorption energy (ΔE_ads_) calculation results for amorphous SiO_2_/Si and crystalline HfO_2_/SiO_2_/Si structure model. **a** From left to right is the model of amorphous SiO_2_/Si structure with single-side interface H passivation, ΔE_ads_ of Si site toward OH species, ΔE_ads_ of Si site toward H_2_O molecule, and ΔE_ads_ of O site toward H_2_O molecule. **b** From left to right is the model of crystalline HfO_2_/SiO_2_/Si structure with single-side interface H passivation, ΔE_ads_ of Hf site toward OH species, ΔE_ads_ of Hf site toward H_2_O molecule, and ΔE_ads_ of O site toward H_2_O molecule


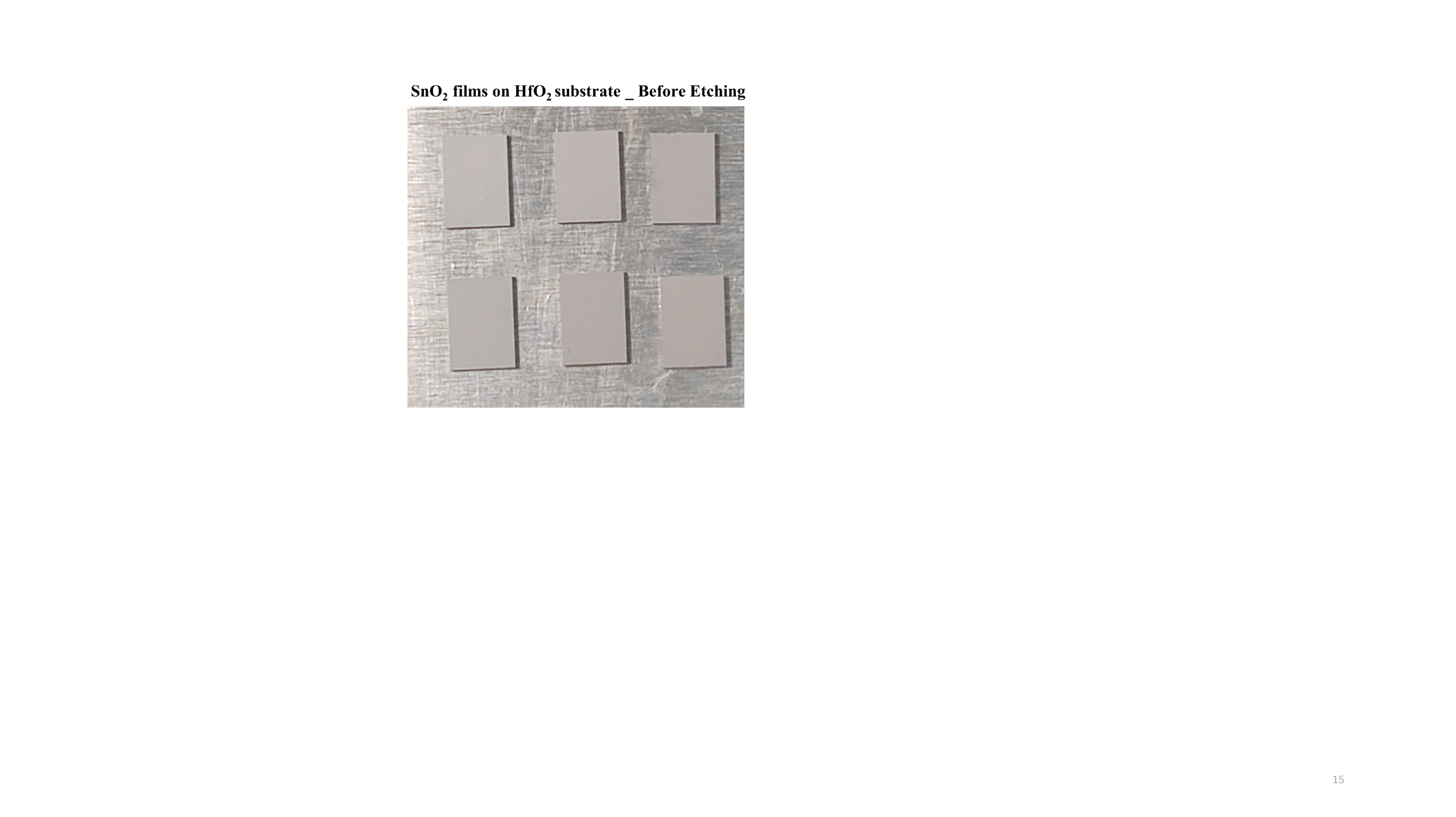


**Fig. S9** Photograph of the self-assembly monolayer SnO_2_ films before wet etching, deposited on a HfO_2_ substrate


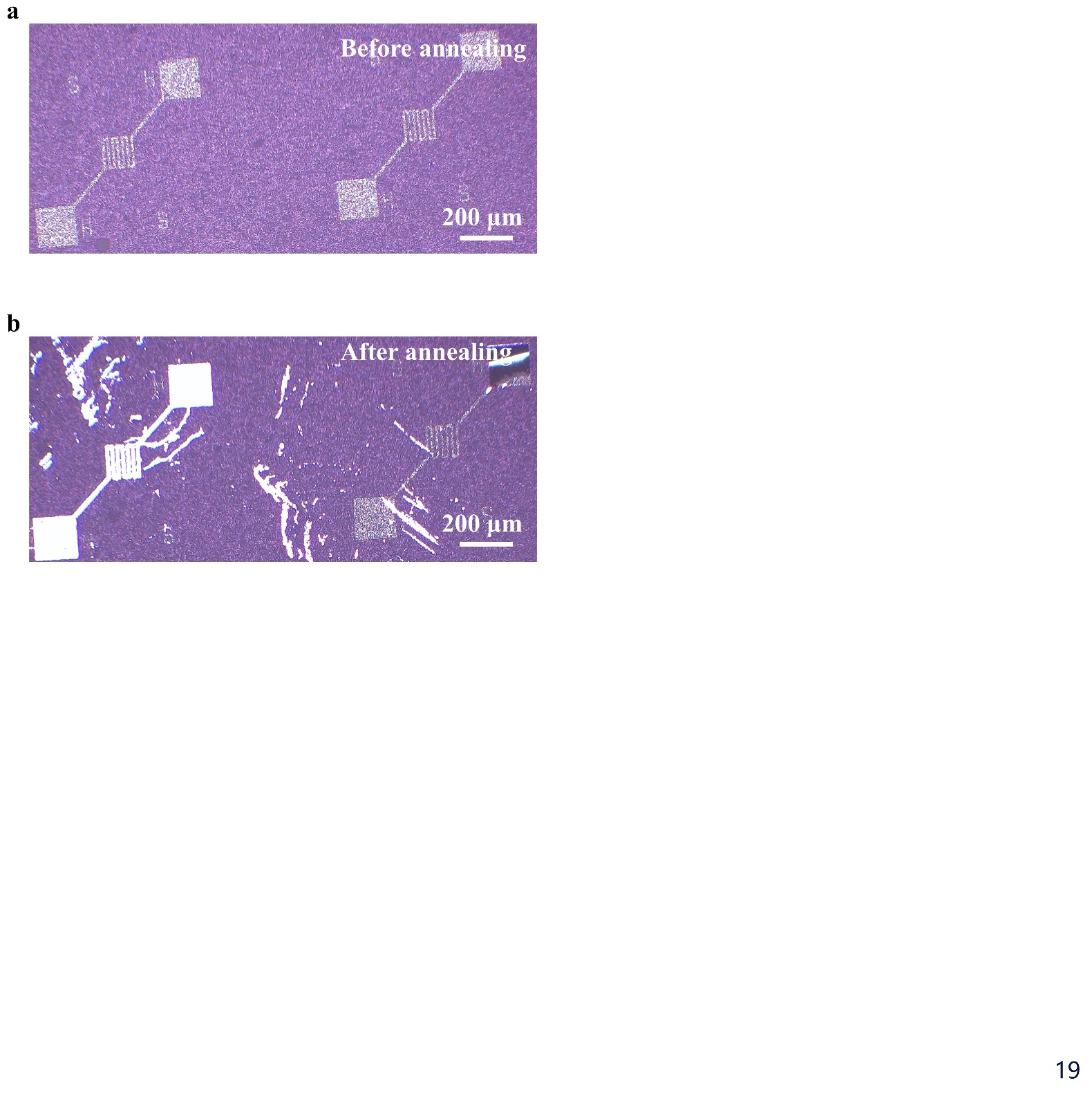


**Fig. S10** Optical microscope images of SnO_2_ film on Ti/Pt electrodes underwent 800 °C annealing. **a** Both SnO_2_ and metal films are intact before annealing. **b** Curvature and detachment occurred in the metal film, leading to the breakage of the SnO_2_ film


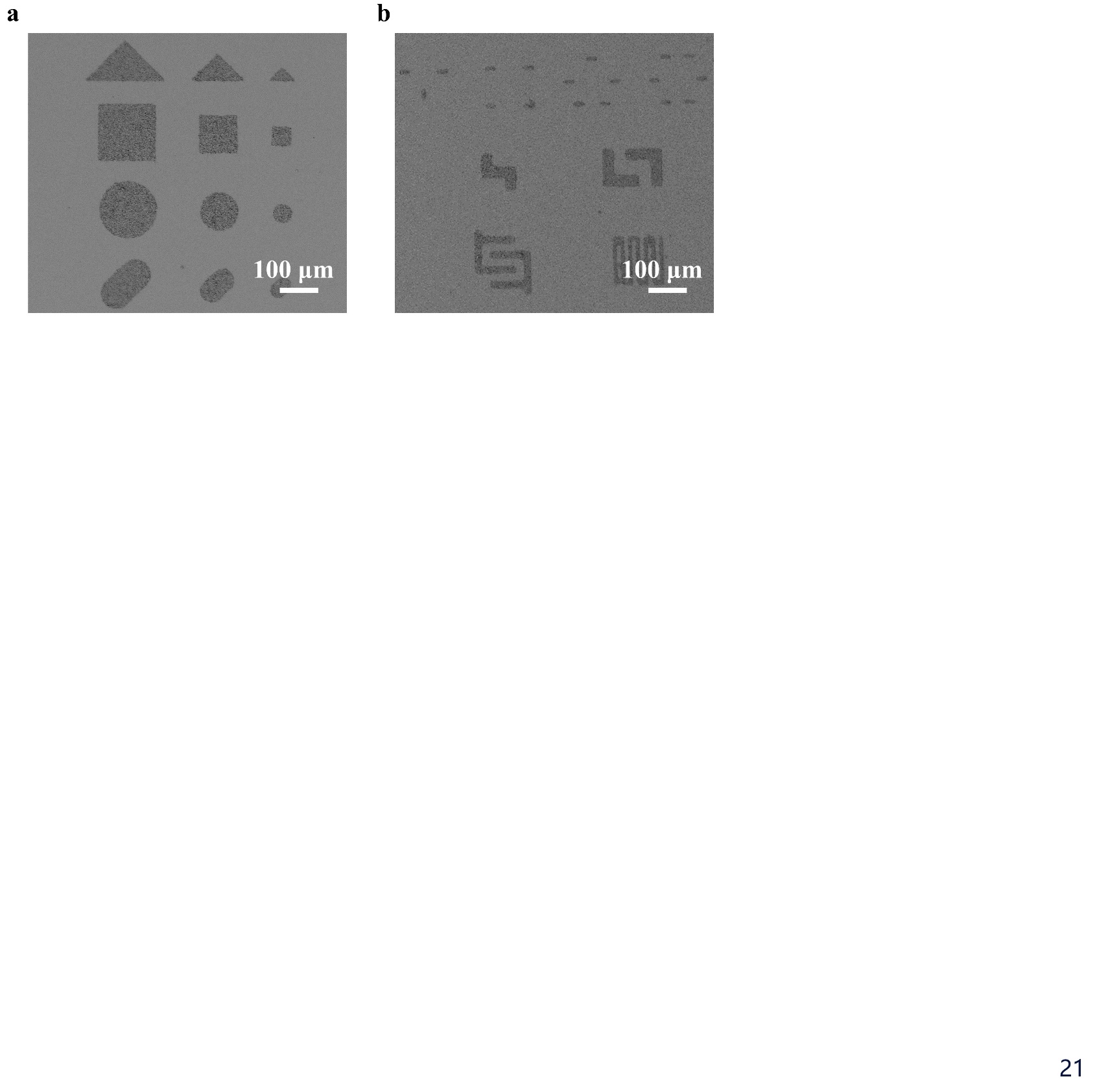


**Fig. S11** SEM images of various SnO_2_ film patterns. **a** The regular shapes are suitable for individual MEMS gas sensors. **b** The irregular shapes can be utilized for MEMS gas sensor arrays structure or more complex sensors structure


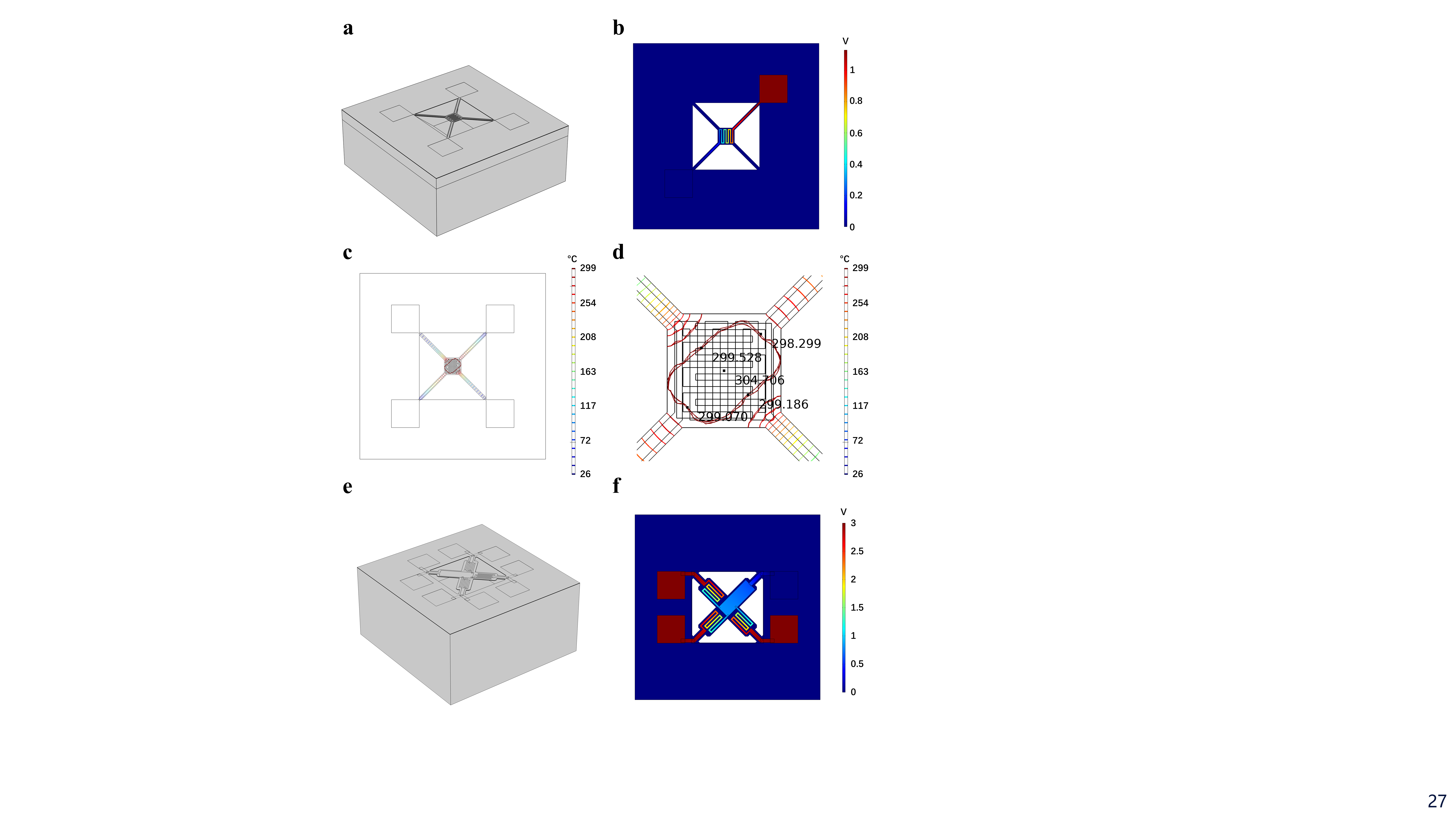


**Fig. S12** Simulation models and results of MEMS hotplates. **a** 3D geometry of MEMS hotplate. **b** Electric field distribution of the heater. **c-d** Thermal distribution and isotherm explanation of MEMS hotplate. **e-f** 3D geometry and electric field distribution of array-type MEMS hotplates


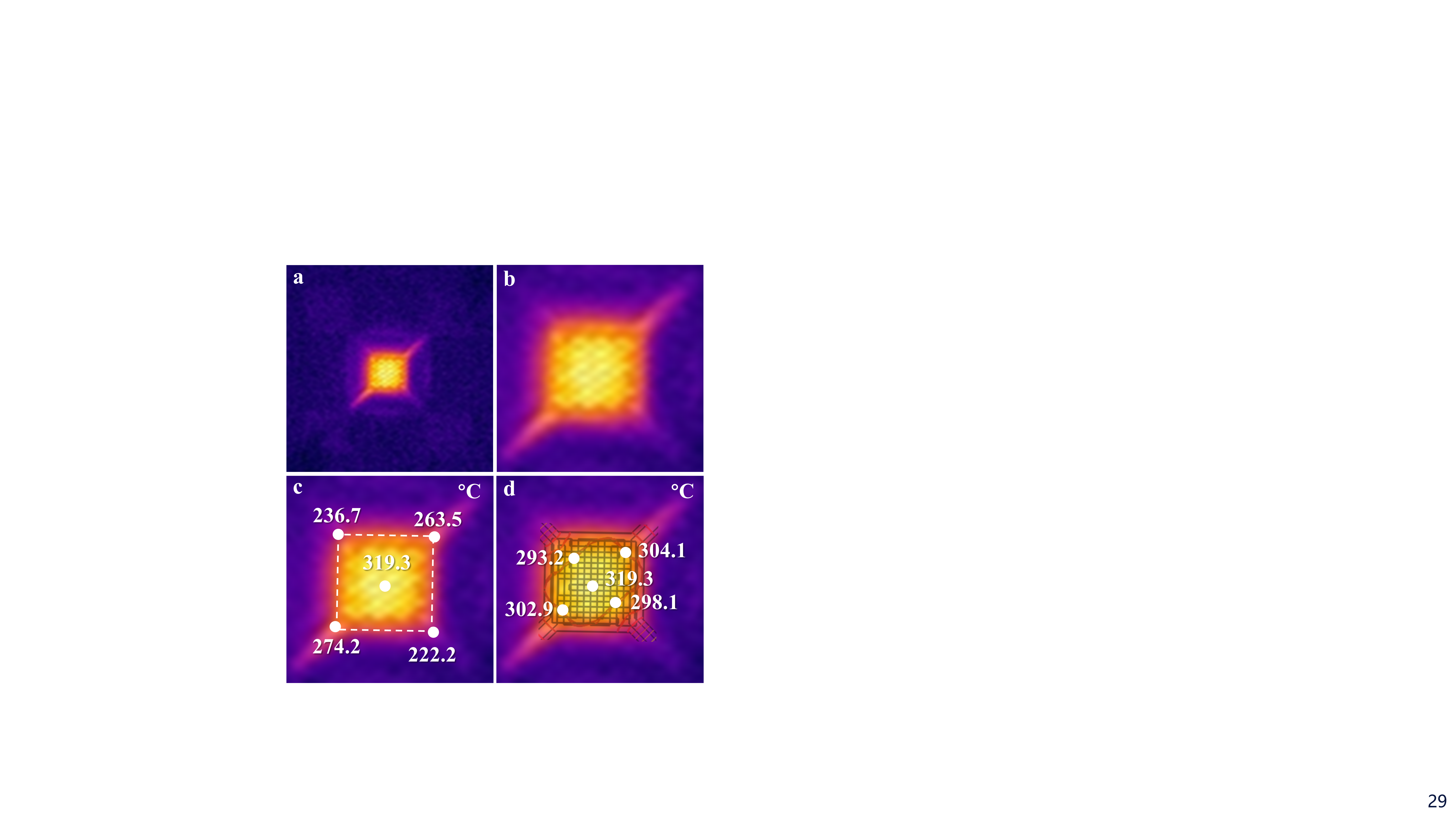


**Fig. S13 a-b** Infrared images of the fabricated MEMS micro hotplate. **c** Thermal distribution of conventional rectangular range of interest. **d** Thermal distribution of isothermal-defined range of interest


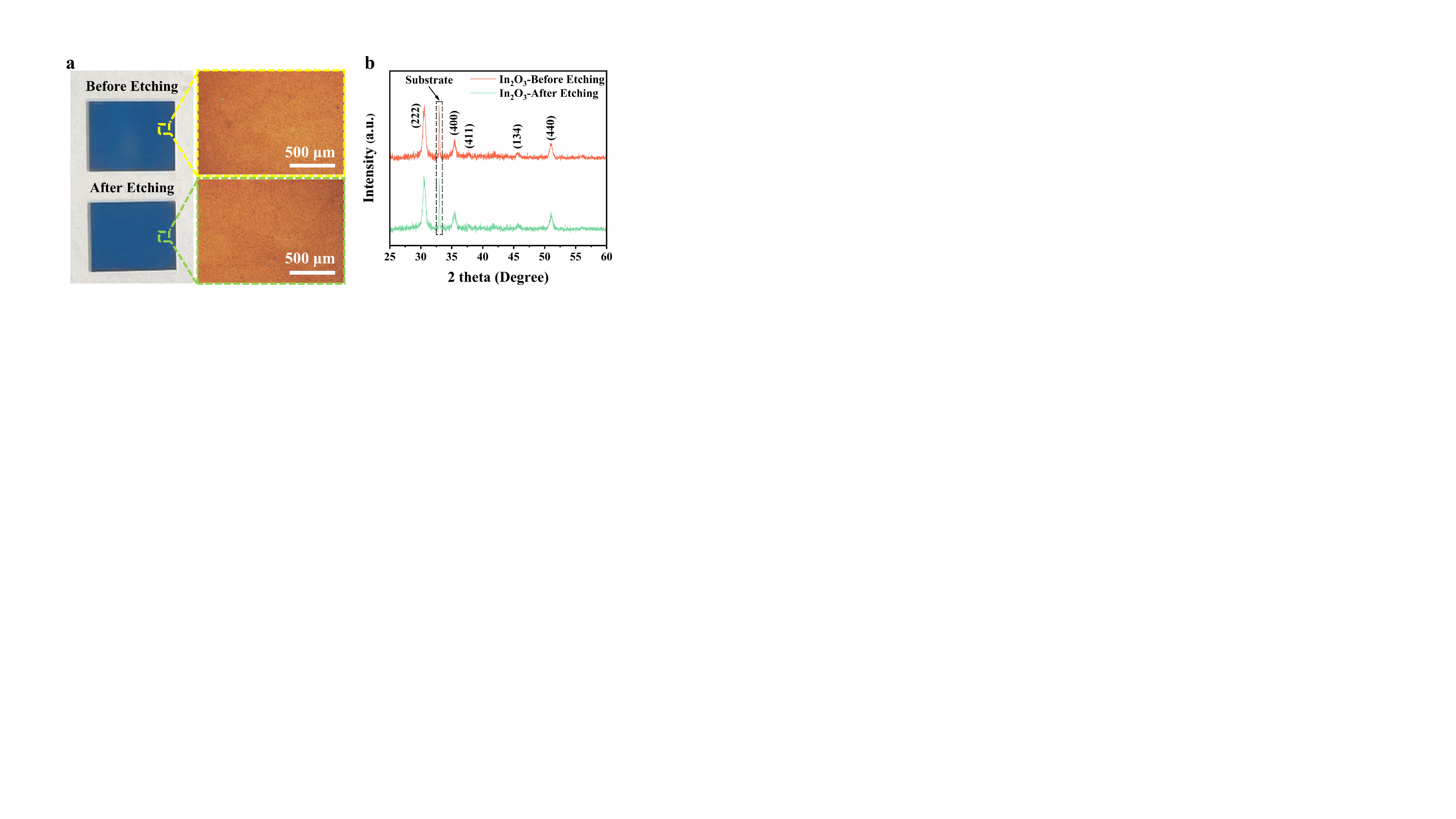


**Fig. S14 a** Comparison of photographs and optical microscope images of In_2_O_3_ films on HfO_2_ substrates before and after etching. **b** Comparison of XRD patterns, both showing the characteristic peaks of In_2_O_3_


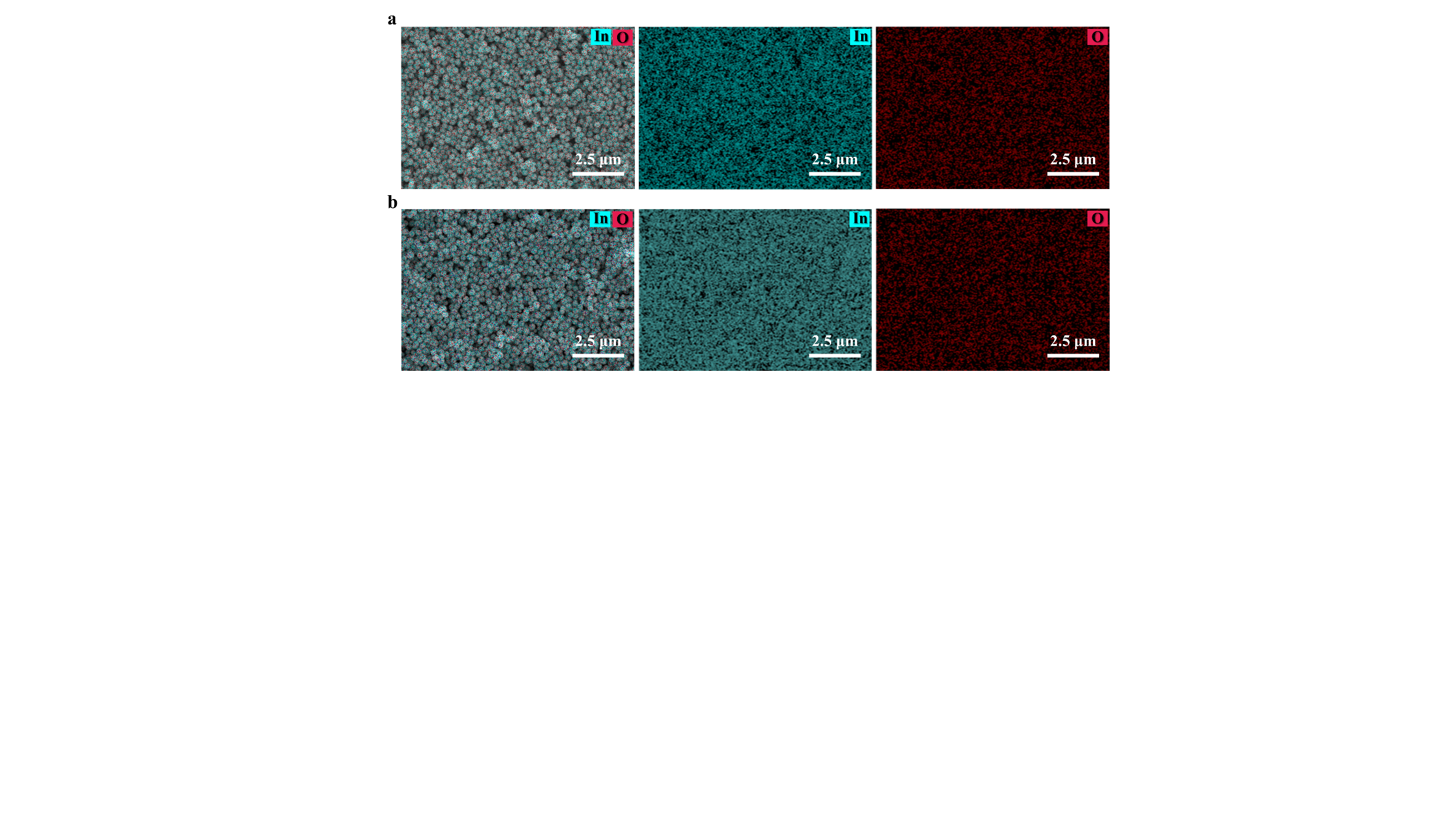


**Fig. S15** Comparison of SEM-EDS images of In_2_O_3_ nanosphere films on HfO_2_ substrates. **a** Before TMAH etching. **b** After TMAH etching. The In_2_O_3_ nanospheres film retains intact


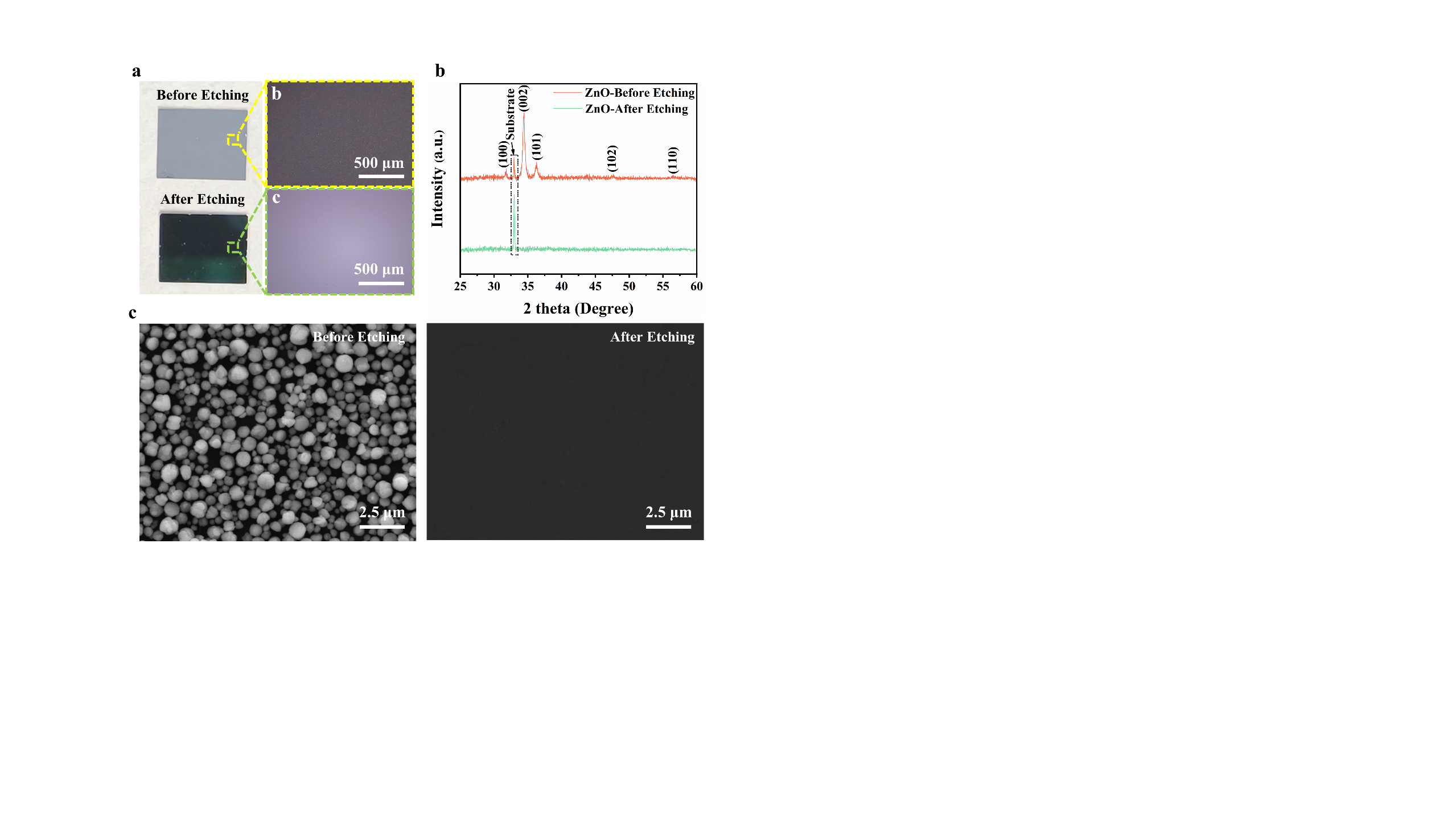


**Fig. S16 a** Comparison of photographs and optical microscope images of ZnO films on HfO_2_ substrates before and after etching. **b** Comparison of XRD patterns, indicating the characteristic peaks of ZnO are absent in the after-etching sample. **c** Comparison of SEM images, highlighting the detachment caused by the etching effect


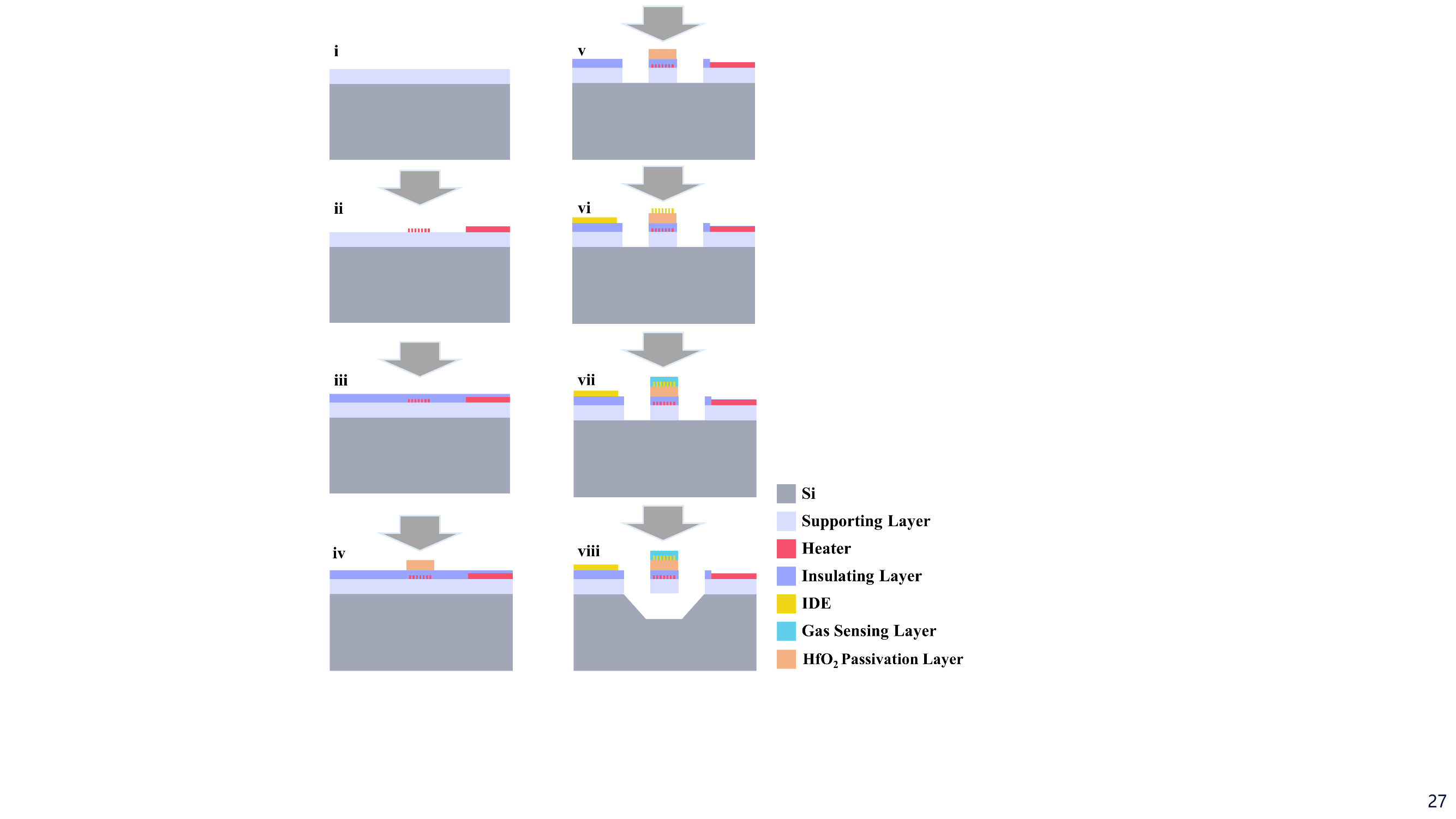


**Fig. S17** Schematic of the complete wafer-level manufacturing process flow for MEMS gas sensing chips. (i) A SiO_2_/Si_3_N_4_/SiO_2_ supporting film is prepared on the Si wafer. (ii) Ti/Pt electrodes are deposited to form the microheaters. (iii) A SiO_2_ insulating layer is deposited. (iv) A HfO_2_ passivation layer is deposited on the central sensing area of the insulating layer. (v) Bonding pads and etching windows are exposed. (vi) Ti/Pt testing electrodes are prepared. (vii) The self-assembly gas sensing film is transferred aligned with HfO_2_. (viii) The suspended cantilever is released by TMAH wet etching, resulting in the final MEMS gas sensing chips


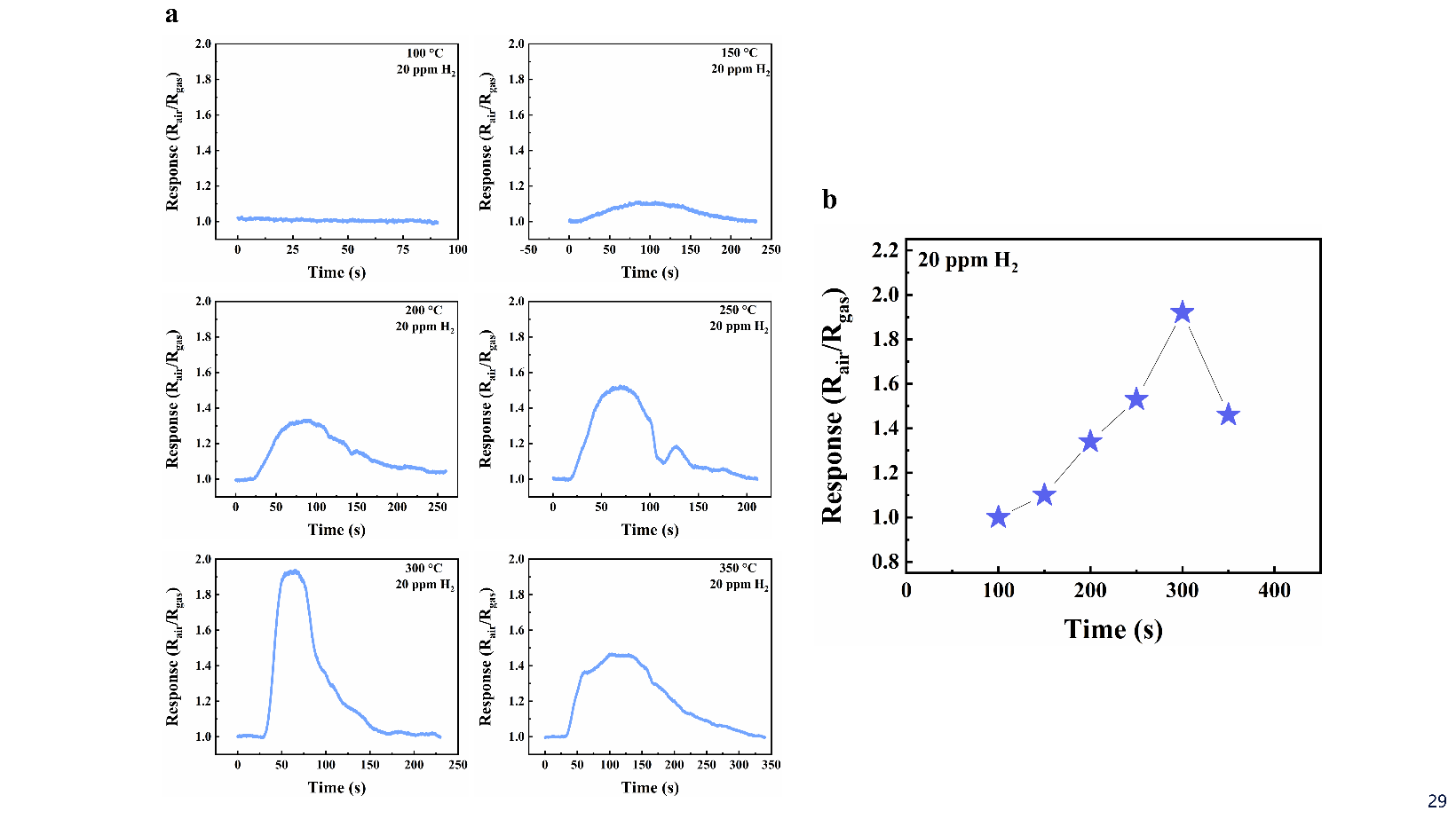


**Fig. S18** Response comparison of Pd/SnO_2_ sensing chips operating at different temperatures


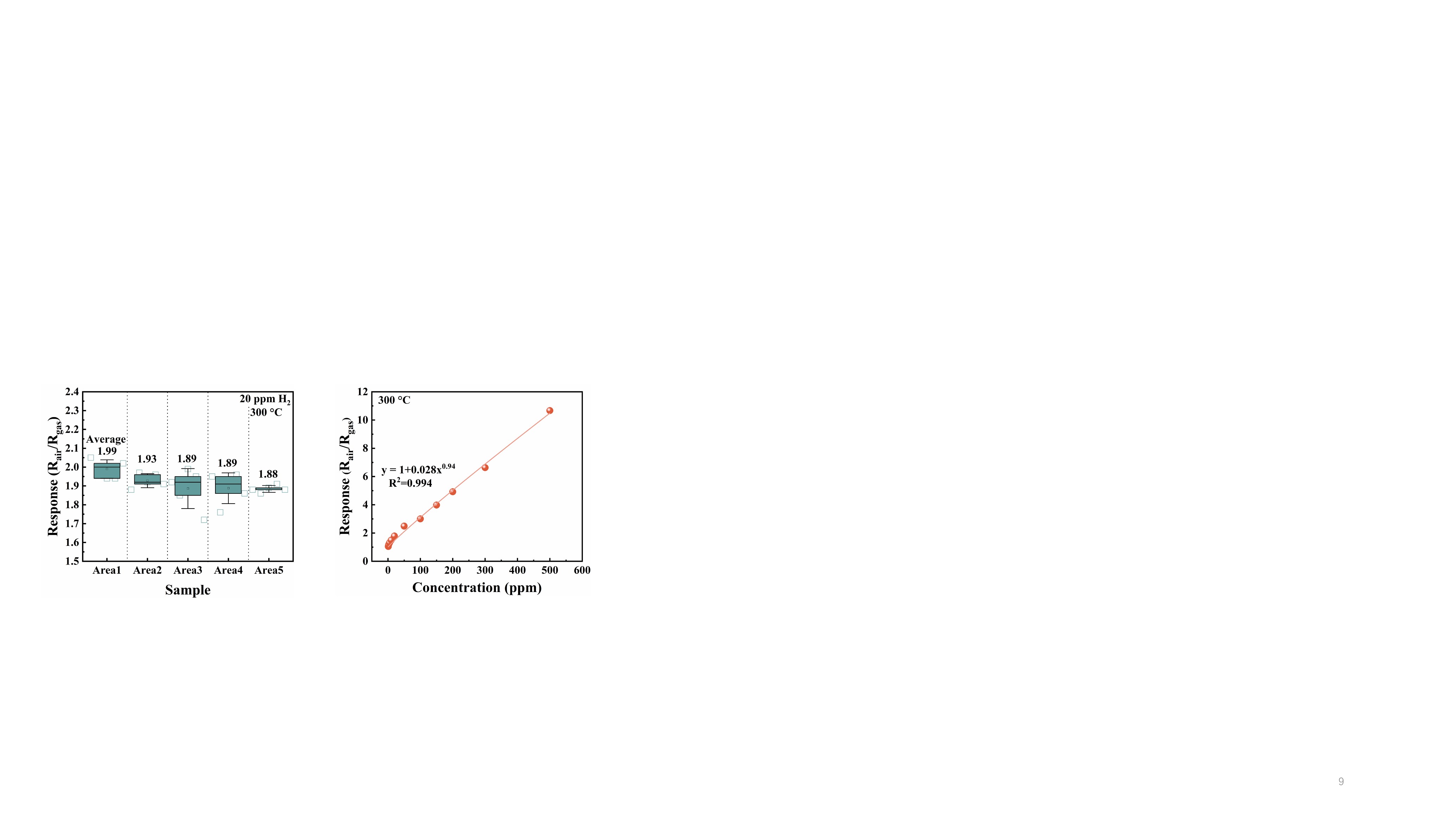


**Fig. S19** Linear fitting curves of the responses of a monolayer Pd/SnO_2_ sensor responses to H_2_ concentrations ranging from 1 to 500 ppm at 300 °C





**Fig. S20** Response time of a monolayer Pd/SnO_2_ sensor towards 20 ppm H_2_ at 300 °C


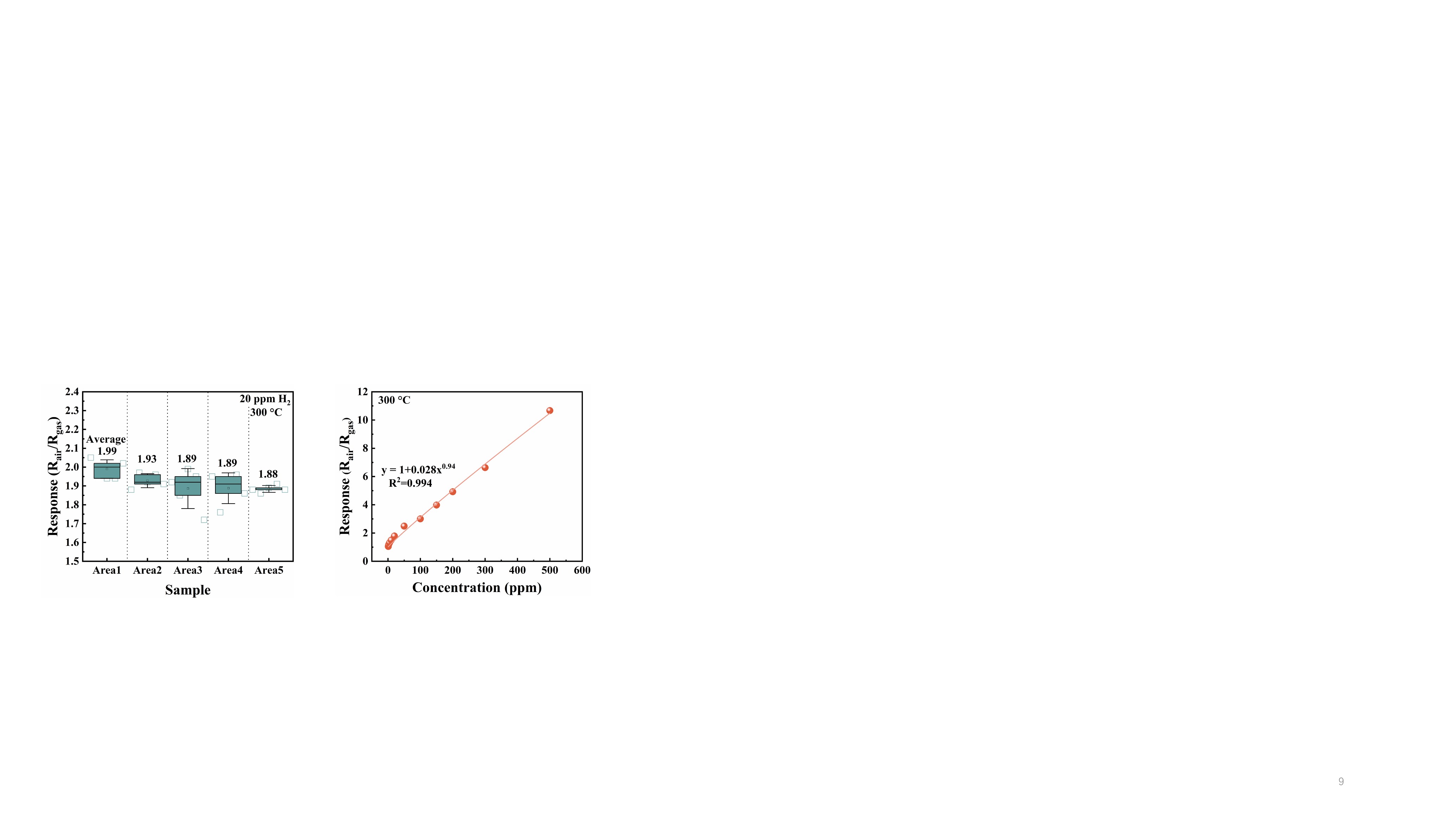


**Fig. S21** Response variation of 25 monolayer Pd/SnO_2_ sensors randomly selected from five different regions of the wafer, with statistical distribution illustrated by box plots


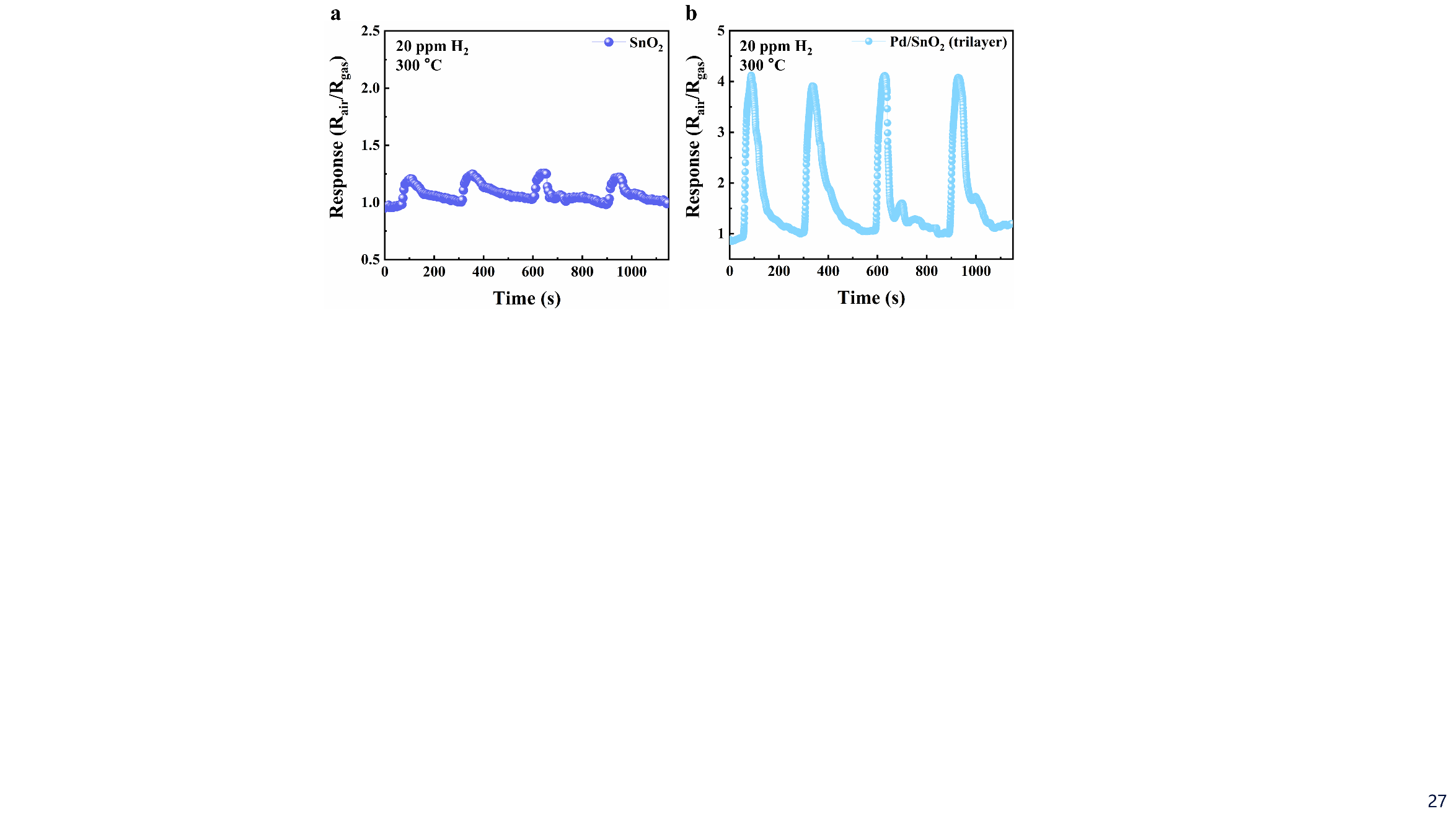


**Fig. S22** **a** Response of monolayer SnO_2_ sensors towards 20 ppm H_2_. **b** Response of trilayer Pd/SnO_2_ sensors towards 20 ppm H_2_

**Table S1** Comparison of fabrication capabilities and H_2_ sensing performance of MEMS micro hotplate sensors with different film-deposition methods

| Sensing materials | Film-deposition methods | Fabrication scale | Film pattern morphology | Concentration Range (ppm) | Response | Response time (s) | Temperature (°C) | Refs. |
| --- | --- | --- | --- | --- | --- | --- | --- | --- |
| Au-Pd dual-metal modified In_2_O_3_ | Dropped by pipette | Single device | Naturally formed | 0.05 ~  100 | 8.35  (10 ppm) | 5.0  (10 ppm) | 245.2 | [S1] |
| (FeCoNi)_100−x_O_x_ | Dropped by needle | Single device | Naturally formed | 200 ~  1000 | 8.60  (200 ppm) | \ | 150.0 | [S2] |
| SnO_2_ | Dropped by pipette | Single device | Naturally formed | 0.1 ~ 20 | 2.21  (6 ppm) | 7.0  (6 ppm) | 250.0 | [S3] |
| Pd NPs/SnO_2_ film | Mask-assistant sputtering and ALD | Wafer level (Silicon) | Rectangle | 0.5 ~  500 | 1.52  (20 ppm) | 115.4  (20 ppm) | 150.0 | [S4] |
| Pd/SnO_2_ nanospheres | Lithography-based self-assembly | Wafer level (Silicon) | Customizable | 1 ~  500 | 1.92*  (20 ppm) | 19.7  (20 ppm) | 300.0 | This work |

* Sensitivity to different H_2_ concentrations at 300 °C: 1.32 (5 ppm), 1.50 (10 ppm), 1.92 (20 ppm), and 4.92 (200 ppm). Sensitivity to 20 ppm H_2_ at different temperatures: 1.00 (100 °C), 1.10 (150 °C), 1.34 (200 °C), 1.53 (250 °C), 1.92 (300 °C), 1.46 (350 °C)

**Supplementary References**

1. Z. Yang, Q. Rong, W. Zhang, X. Fan, M. Shao et al., A ZnO packaged MEMS hydrogen sensor for reliable sibo breath analysis with anti-H2S interference. Chem Eng J (Lausanne). **522**, 167331 (2025). https://doi.org/10.1016/j.cej.2025.167331
2. W. Yan, Y. Liu, Y. Bai, Y. Chen, H. Zhou et al., Intelligent MEMS sensor based on an oxidized medium-entropy alloy (FeCoNi) for H_2_ and co recognition. ACS Appl Mater Interfaces. **16**(37), 49474-49483 (2024). <https://doi.org/10.1021/acsami.4c07782>
3. N. Luo, C. Wang, D. Zhang, M. Guo, X. Wang et al., Ultralow detection limit MEMS hydrogen sensor based on SnO_2_ with oxygen vacancies. Sens Actuators B Chem. **354**, 130982 (2022). https://doi.org/10.1016/j.snb.2021.130982
4. Z. Zhang, L. Luo, Y. Zhang, G. Lv, Y. Luo et al., Wafer-level manufacturing of MEMS H_2_ sensing chips based on Pd nanoparticles modified SnO_2_ film patterns. Adv Sci. **10**(26), 2302614 (2023). https://doi.org/10.1002/advs.202302614W

ss
